# Biopharmaceutical Characterization and Stability of Nabumetone–Cyclodextrins Complexes Prepared by Grinding

**DOI:** 10.3390/pharmaceutics16121493

**Published:** 2024-11-21

**Authors:** David Klarić, Željka Soldin, Anna Vincze, Rita Szolláth, György Tibor Balogh, Mario Jug, Nives Galić

**Affiliations:** 1 Department of Chemistry, Faculty of Science, University of Zagreb, Horvatovac 102a, 10 000 Zagreb, Croatia; dklaric@chem.pmf.hr (D.K.); zeljka@chem.pmf.hr (Ž.S.); 2 Department of Pharmaceutical Chemistry, Semmelweis University, Hőgyes Endre u. 9., H-1092 Budapest, Hungary; vincze.anna@semmelweis.hu (A.V.); szollath.rita.mariann@semmelweis.hu (R.S.); balogh.gyorgy.tibor@semmelweis.hu (G.T.B.); 3 Center for Pharmacology and Drug Research & Development, Semmelweis University, Üllői u. 26. H-1092 Budapest, Hungary; 4 Department of Pharmaceutical Technology, Faculty of Pharmacy and Biochemistry, University of Zagreb, A. Kovačića 1, 10 000 Zagreb, Croatia

**Keywords:** nabumetone, cyclodextrins, mechanochemistry, solid-state characterization, in vitro permeability, in vitro dissolution, degradants, chemical stability

## Abstract

**Background:** Nabumetone (NAB) is a poorly soluble nonsteroidal anti-inflammatory prodrug (BCS class II drug) whose solubility is significantly improved by complexation with cyclodextrins (CDs). **Methods**: The solid complexes, in a 1:1 molar ratio, were prepared by mechanochemical activation by grinding, using β-cyclodextrin (β-CD) and its derivatives, hydroxypropyl- and sulfobutylether-β-cyclodextrin (HP-β-CD and SBE-β-CD). The complexation was confirmed by differential scanning calorimetry (DSC), powder X-ray diffraction (PXRD), and attenuated total reflectance Fourier-transformed infrared spectroscopy (ATR–FTIR). Obtained products were further characterized regarding their solubility, in vitro dissolution, permeability and chemical stability. **Results**: Co-grinding with HP-β-CD and SBE-β-CD yielded products that showed in vitro dissolution profiles in hydrochloric acid medium (pH 1.2) that were substantially different from that of pure NAB, yielding dissolution efficiency enhancements of 34.86 ± 1.64 and 58.30 ± 0.28 times, respectively, for the optimized products. Their in vitro dissolution and gastrointestinal permeability were also studied in a low-volume environment at pH 6.8, corresponding to the intestinal environment. Both β-CD derivatives increased NAB dissolution rate and NAB mass transport across the biomimetic membrane. The effect of β-CD derivatives on NAB chemical stability was studied under the stress conditions by the developed and validated UHPLC–DAD–HRMS method. In acidic conditions, pure and complexed NAB was prone to hydrolytic degradation, yielding one degradation product—pharmacologically inactive NAB metabolite. However, under the oxidative conditions at elevated temperatures, 10 NAB degradation products were identified from co-ground samples. All systems were stable during photo- and long-term stability studies. **Conclusions**: NAB complexes with HP-β-CD and SBE-β-CD are promising candidates for pharmaceutical product development.

## 1. Introduction

Developing a new drug formulation is a demanding and complex procedure which needs a detailed characterization of the system both in solution and in the solid state. Cyclodextrins (CDs), a versatile family of cyclic oligosaccharides with a unique structure, have gathered significant interest as excipients due to their potential to form inclusion complexes [1]. These complexes can significantly enhance the solubility, chemical stability, and bioavailability of various drugs [2,3,4]. The biocompatibility, good tolerability, non-immunogenicity, and functional versatility make CDs a valuable asset in pharmaceutical formulations, particularly for newly emerging drug candidates with increasing molecular mass, lipophilicity, and reduced water solubility [5]. They also offer a solution for reformulating existing drugs with limited aqueous solubility, avoiding potentially irritant excipients like cosolvents and surfactants, resulting in formulations with enhanced tolerability and patient acceptance [6,7]. Numerous pharmaceutical products based on CDs have been subjected to clinical trials or have already reached the global market [2]. 

Very recently, within the ongoing project on drug–CDs complexes with enhanced properties, we have presented the results of a comprehensive investigation of nabumetone/CDs inclusion complexes in solution [8]. Since β-cyclodextrin (β-CD) and its derivatives increased nabumetone (NAB) solubility due to the formation of 1:1 inclusion complexes, in the continuation of our study on improving the solubility of poorly soluble drugs [9,10], in this work, the preparation and characterization of NAB complexes in the solid state is presented. 

Solid drug/CD complexes, the starting material for dosage form development, may be prepared from solution through crystallization, spray-drying, and lyophilization; in the semisolid state by kneading; and in the solid state [11]. Considering the rising importance of green chemistry approaches, solid-state methods are of great interest, as they eliminate problems related to the low solubility and chemical instability of drugs in solution, as well as the potential toxic effect of residual solvents. In this light, mechanochemical activation by grinding appears as a fast, highly efficient, solvent-free, sustainable approach [12,13]. Furthermore, the grinding process may raise unconventional supramolecular interactions that lead to novel molecular arrangements not observed in solution, providing significant advantages in the formulation development [14]. 

**Figure 1:**
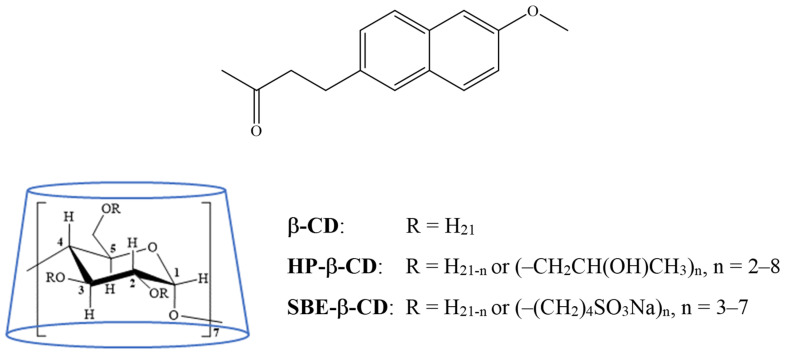
Structural formulas of nabumetone (NAB), β-cyclodextrin (β-CD), and their derivatives.

Considering all the above, we applied mechanochemical activation by grinding to prepare solid CD complexes of NAB, a poorly soluble nonsteroidal anti-inflammatory prodrug (Figure 1). NAB exhibits a pharmacological effect via the metabolite 6-methoxy-2-naphthylacetic acid and is administered orally to treat patients with osteoarthritis and rheumatoid arthritis, showing comparable efficiency and better gastrointestinal tolerability than other nonsteroid anti-inflammatory drugs [15,16]. While the interactions of CDs and NAB have been extensively studied in solution [8,17,18,19], the systematic screening of preparation methods for NAB/CDs complexes in the solid state represents a novel and intriguing area of research. Previous studies have focused on methods like co-evaporation, kneading, and coprecipitation [20], which are suitable for laboratory-scale preparation but have limited potential for industrial application. In this study, a series of products were prepared by grinding in a high-energy vibrational mill that employed different CD derivatives and processing parameters to develop a product suitable for further formulation development. With that aim, we have screened the efficiency of nabumetone co-grinding with β-cyclodextrin (β-CD) as well as hydroxypropyl- and sulfobutylether-β-cyclodextrin (HP-β-CD and SBE-β-CD, respectively) by changing the grinding time and the grinding frequency. Complexes were prepared at an equimolar drug-to-cyclodextrin ratio, considering the results of a previous study performed by our group [8]. Differential scanning calorimetry (DSC), powder X-ray diffraction (PXRD), and attenuated total reflectance Fourier-transformed infrared spectroscopy (ATR–FTIR) were used to monitor the solid-state interactions in the products prepared. Obtained products were further characterized regarding their solubility, in vitro dissolution in the simulated gastric medium, and in vitro permeability, and flux was analyzed using a MicroFLUX device equipped with an artificial biomimetic membrane. To assess the stability profile of the cyclodextrin complexes obtained, forced degradation studies, employing oxidative, acidic, and basic conditions, were performed. The structures of degradants were proposed based on the results obtained using ultra-high-performance liquid chromatography coupled with high-resolution mass spectrometry (UHPLC–HRMS). This enabled the performance of the photostability and the long-term stability studies according to the ICH guidelines that, combined with other results, enabled the critical evaluation and selection of the complexes that were suitable for further formulation development. 

## 2. Material and Methods

### 2.1. Materials

Nabumetone (NAB) was purchased from Cayman Chemical (Ann Arbor, MI, USA). β-cyclodextrin (β-CD), 2-hydroxypropyl-β-cyclodextrin (HP-β-CD, with an average degree of substitution, DS = 4.5), and sulfobutylether sodium salt β-cyclodextrin (SBE-β-CD, DS = 6.5) were obtained from CycloLab (Budapest, Hungary). Methanol and formic acid, LC–MS grade, were purchased from Carlo Erba (Milano, Italy). Ultrapure water was obtained from a Mili-Q Advantage A10 purification system (Merck, Darmstadt, Germany). All other chemicals were of p.a. grade and used as received.

Hydrochloric acid medium (pH 1.2) and hydrochloric medium (pH = 1.2) containing 2% (*w*/*V*) sodium lauryl sulfate were prepared according to European Pharmacopoeia [21].

Analytical-grade solvents (hexane, dodecane, chloroform), L-α-phosphatidylcholine, cholesterol, and all chemicals used for the buffer preparation were purchased from Merck KGaA (Darmstadt, Germany). Phosphate-buffered saline solution (PBS solution; 0.01 M, pH 6.8 and pH 7.4) was prepared by mixing 0.01 M monosodium phosphate solution (0.01 M NaH_2_PO_4_, 0.0027 M KCl, 0.138 M NaCl) and 0.01 M disodium phosphate solution together (0.01 M Na_2_HPO_4_, 0.0027 M KCl, 0.138 M NaCl), adjusting the pH with HCl. The phospholipid solution needed for flux measurements was prepared by dissolving 16 mg phosphatidylcholine and 4 mg cholesterol in 600 μL solvent mixture (70:25:5 *V/V* hexane:dodecane:chloroform).

### 2.2. Complex Preparation in the Solid State 

#### 2.2.1. Complex Preparation by Grinding

Cyclodextrin complexes of NAB were prepared by neat-grinding (NG) in a high-energy vibrational ball mill (Mixer Mill MM 500 control, Retch, GmbH, Germany), employing β-CD, HP-β-CD, and SBE-β-CD in equimolar ratios to the drug, in line with previous results [8]. Then, 6 g sample batches were prepared using 50 mL grinding jars coated with ZrO_2_, equipped with 15 5 mm and 15 10 mm grinding balls made of the same material. The grinding was performed at a controlled temperature of 20 °C, applying the different grinding periods of up to 120 min in cycles comprising 1 min grinding at 5 Hz to homogenize the product followed by 15 min grinding at a frequency of 20 or 30 Hz to achieve complexation. The drug alone was also subjected to the same procedure at 20 Hz to evaluate the effect of mechanochemical activation on the physicochemical properties of the drug. The obtained products were transferred to the airtight glass containers and stored in a desiccator at room temperature until further analysis.

#### 2.2.2. Complex Preparation by Co-Evaporation

Cyclodextrin complexes of NAB were also prepared by co-evaporation, according to the previously described method [20]. Briefly, 0.05 g of NAB was dissolved in 20 mL of concentrated ethanol, while an equimolar amount of CD in question was dissolved in the same volume of purified water. Then, both solutions were mixed, and the solvent was removed using an IKA KS 4000i control thermostated orbital shaker (IKA-Werke GmbH & Co., Staufen, Germany) operating at 50 °C with gentle mixing at 100 rpm. The drug alone was also subjected to the same procedure by omitting CDs from the preparation to evaluate the effect of co-evaporation on the physicochemical properties of the drug. The obtained products were transferred to the airtight glass containers and stored in a desiccator at room temperature until further analysis.

### 2.3. Solid State Analysis

Differential scanning calorimetry (DSC) was performed on a Perkin-Elmer Diamond differential scanning calorimeter (PerkinElmer, Inc., Waltham, MA, USA) calibrated with indium (99.98% purity; melting point 156.61 °C and fusion enthalpy of 28.71 J/g). The samples were accurately weighted into aluminum pans (1–3 mg, Mettler M3 Microbalance, Germany), sealed with pierced lids, and scanned under the inert atmosphere (nitrogen purge of 25 mL/min), employing a heating rate of 10 °C/min over the temperature range of 25–120 °C. The relative degree of drug crystallinity (*RDC*) in the samples was calculated according to Equation (1):

(1) $$RDC=\frac{\Delta H_{sample}}{\Delta H_{drug}}\times 100\%$$

where Δ*H*_sample_ and Δ*H*_drug_ are the fusion enthalpies of the NAB in the product (normalized to the drug content) and the pure drug, respectively. Measurements were carried out in triplicate, and the relative standard deviation of crystallinity data was <1.0%.

Powder X-ray diffractograms (PXRD) of the starting compounds and co-ground products were recorded using a Malvern Panalytical Aeris diffractometer with Bragg–Brentano geometry (Malvern Panalytical LTD, Malvern, UK) with a copper anode (CuKα, 1.5406 Å) as a radiation source, a Ni filter, and a PIXcel1D-Medipix3 detector at room temperature on a silicon support. The intensity of the diffracted radiation was recorded by continuous scan in the range of 2θ values from 5 to 50° employing the scanning speed of 0.022 °/s, where the counter remained at a single point for 18.87 s. The Kα1/Kα2 intensity ratio was 0.5, the working voltage of the tube was 40 kV, and the cathode was heated with a current of 15 mA. 

Attenuated total reflection infrared spectra (ATR–FTIR) spectra of the samples were recorded in the wave number range 4000–400 cm^–1^, with an optical resolution of 2 cm^–1^, using a Bruker Vector 22 IR spectrometer (Bruker Optics GmbH, Ettlingen, Germany) equipped with a Pike MIRacle ATR mount with diamond/ZnSe surface. All measurements were performed at ambient conditions by averaging 32 independent scans. The obtained spectra were further processed using the Opus 6.0 computer program, which applied ATR and background correction. Finally, the spectra were smoothed with the Savitzky–Golay algorithm through 25 data points.

### 2.4. Solubility and In Vitro Dissolution Study at pH 1.2

The saturated solubility of NAB and prepared co-ground products with CDs was determined by adding 30 mg of the drug or the equivalent amount of co-ground CD complex to 15 mL of the medium, which included a hydrochloric acid medium (pH = 1.2), a hydrochloric medium (pH = 1.2) containing 2% (*w*/*V*) sodium lauryl sulfate, and a series of aqueous methanolic solutions (5–40%, *V*/*V*). The flasks were hermetically sealed and shaken at 120 rpm in an orbital shaker thermostated at 37 °C (IKA^®^ KS 4000 i control, IKA-Werke GmbH & Co. KG, Staufen, Germany). After 24 and 48 h, the sample aliquots were filtered using Cromafil^®^Xtra PES 20/25 membrane filter (Macherey-Nagel GmbH & Co. KG, Düren, Germany) and spectrophotometrically assayed at *λ* = 230 nm (Agilent Carry 60 spectrophotometer Agilent Technologies, Inc. Santa Clara, CA, USA) to determine the amount of dissolved drug [22]. Preliminary studies demonstrated that NAB did not adsorb to the filters used to separate the undissolved solids. The experiment was performed in triplicate for each sample.

The in vitro dissolution of NAB from the prepared solid systems was investigated using the mini paddle setup of the Agilent 708-DS Dissolution Apparatus (Agilent Technologies, Inc., USA). The sample quantity containing 250 mg of NAB was added to 125 mL of the dissolution medium, thermostated at 37 °C, and stirred at 100 rpm. At preselected time intervals (2, 5, 10, 15, 20, 30, 45, and 60 min), 5 mL aliquots of the dissolution medium were sampled and immediately replaced with the same volume of fresh medium, thermostated at 37 °C, to provide a constant volume of the dissolution medium during the experiment. Collected aliquots were filtered through Cromafil^®^Xtra PES 20/25 membrane filter (Macherey-Nagel GmbH & Co. KG, Düren, Germany) with 0.20 μm pore size and spectrophotometrically assayed for drug content as described above. The test was performed in triplicate (CV < 5.0%) for each sample. A correction was applied for the cumulative dissolution caused by adding the fresh dissolution medium. 

The obtained in vitro dissolution profiles were characterized by calculating the dissolution efficiency (*DE*) parameter according to Equation (2):

(2) $$DE_{60\;min}=\frac{\int_{0}^{t}Qdt}{Q_{100\%}\times t}\times 100$$

where *Q* is the percentage of the dissolved drug and *t* is the examined dissolution time [23]. Furthermore, a model-independent similarity factor *f*_2_ was used to compare the in vitro dissolution profiles, calculated according to Equation (3):

(3) $$f_{2}=50\times log\left\{{\left[1+\frac{1}{n}\overset{n}{\underset{t=1}{\sum }}{\left(R_{t}-T_{t}\right)}^{2}\right]}^{-0.5}\times 100\right\}$$

where *n* is the number of time points and *R*_t_ and *T*_t_ are the dissolution value of the reference and test product, respectively, at time *t* [24].

### 2.5. In Vitro Assessment of Dissolution–Absorption Processes in a Low-Volume Environment

#### 2.5.1. In Vitro Dissolution Study at pH 6.8

For the in vitro dissolution studies, 20 mg of the samples (NAB, NAB/HP-β-CD GR 30 Hz/90 min, NAB/SBE-β-CD GR 30 Hz/120 min) were placed into small vessels containing 20 mL PBS solutions (pH 6.8, modeling the intestinal fluid). Measurements were carried out in triplicate. The suspensions were stirred at 300 rpm for only the first 6 h; then, they were allowed to settle for the remaining time. UV spectra were recorded by immersing the UV probes of a Rainbow R6 dynamic dissolution monitor (pION Inc., Billerica, MA, USA) over 24 h. The thermodynamic solubility of each sample was determined as the equilibrium concentration reached in this time period.

#### 2.5.2. In Vitro Gastrointestinal Permeability and Flux Measurements

The flux of nabumetone in different formulations was measured using a MicroFLUX device (pION Inc., USA), consisting of two low-volume absorption chambers and a separating PVDF membrane disk (0.45 μm pore size, 1.54 cm^2^). First, the membrane disk was wetted with 20 μL phospholipid solution to create a gastrointestinal biomimetic membrane. The donor chamber was filled with 20 mL PBS pH 6.8 solution. Meanwhile, the acceptor chamber was filled with the same amount of 1 mM SBE-β-CD solution (dissolved in PBS pH 7.4) to ensure sink conditions throughout the measurement. Then, a magnetic stirrer bar was placed in both chambers. At the start of the experiment, 20 mg of the sample (NAB, NAB/HP-β-CD GR 30 Hz/90 min, NAB/SBE-β-CD GR 30 Hz/120 min) was added to the donor chambers, and the stirring was immediately started. All measurements were carried out in triplicate at 37 °C and 300 rpm stirring. The UV–Vis data of each chamber were recorded by immersing the UV probes of a Rainbow R6 dynamic dissolution monitor (pION Inc., USA) over a 4-hour period.

Flux was calculated using Equation (4):

(4) $$J\left(t\right)=\frac{\Delta n}{\times \Delta t}$$

where *J* is the flux $\left(\frac{\mathrm{mol}}{{\mathrm{cm}}^{2}}\cdot s\right),\;$*n* is the amount of drug crossing the membrane (mol), *A* is the area of the membrane (1.54 cm^2^), and *t* is the time (s).

Permeability was calculated using Equation (5):

(5) $$P_{a}=\frac{J\left(t\right)}{c_{D}\left(t\right)}$$

where $P_{a}$ is the apparent permeability (cm/s) and $c_{D}\left(t\right)$ is the donor concentration at 4 h (mol/cm^3^).

#### 2.5.3. UV–Vis Spectroscopy

The concentrations of NAB in the in vitro solubility (pH 6.8) and flux measurements were determined using a Rainbow R6 dynamic dissolution monitor (pION Inc., USA). The six UV–Vis immersion probes were equipped with 10 mm pathlength tips. Spectra were recorded in the 220–500 nm wavelength range. Concentration was calculated using the AuPRO 7.1 software (pION Inc., USA) based on a calibration curve generated prior to the measurements.

### 2.6. Stability Testing of Prepared Cyclodextrin Complexes

#### 2.6.1. Stress Conditions

A stock solution of NAB was prepared in MeOH, LC–MS grade (1 mg/mL), while those of the CD complexes were prepared in a 1:1 mixture of MeOH and H_2_O (1 mg/mL). Forced degradation was performed under the following conditions: pure water, 2 M HCl, 2 M NaOH, and 3% H_2_O_2_ at 80 °C for 10 h and, in the case of oxidative degradation, at room temperature for 7 days. 

#### 2.6.2. Photostability Study

NAB and the complexes were sieved in glass petri dishes (*ϕ* = 50 mm) to produce a 1 mm thin layer. The control samples were prepared in the same way, but the petri dish was covered and wrapped with aluminum foil. All samples were put in stability chambers and exposed for 141.2 h to a daylight source (8.5 Klux) and 40 h to a UV source (5 W/m^2^) to ensure that standard ICH Q1B photostability testing conditions were achieved, i.e., 1,200,000 lux h and 200 W H/m^2^ [25]. 

#### 2.6.3. Long-Term Stability

NAB and co-ground NAB/CD solids (200 ± 50 mg) were added to Eppendorf tubes and placed in a stability chamber (Memmert ICH110L eco) at 25 °C and 60% relative humidity (RH). Samples were collected and analyzed after 0, 1, 2, 3, 6, and 9 months. Next, 5 mg of NAB (10 mg of the complex) was dissolved in 5 mL of MeOH in the 10 mL flask using an ultrasonic bath. Water was then added until the volume reached the mark. The mass of the samples was corrected for the moisture content determined by dynamic thermogravimetric measurement at 10 °C/min using Discovery TGA 550 (TA Instruments, New Castle, DE, USA). All samples were analyzed in triplicate. 

### 2.7. Chromatographic and MS/MS Measurements

Stability-indicating UHPLC–DAD (Agilent 1290 infinity II) and UHPLC–HRMS (Agilent 6550 iFunnel Q-TOF) methods were developed and validated for the quantitative determination of NAB and its degradation products (Supporting Information, Tables S1 and S2). Chromatographic separation was achieved on Agilent ZORBAX RRHD Eclipse Plus C18 column (2.1 × 50 mm, 1.8 µm) in gradient mode using a mobile phase consisting of 0.1% formic acid in water (solvent A) and in methanol (solvent B), with the flow rate set to 0.20 mL/min. The injection volume was 1 µL. NAB was detected at 231 nm. MS/MS experiments were conducted in ion-positive mode using N_2_ as a collision gas. All samples were filtered through a Chromafil O-20/15 MS filter before LC–DAD and LC–MS analyses. The NAB concentration in MeOH:H_2_O 1:1 working solutions amounted to 2 µg/mL. 

### 2.8. Statistical Analysis

Data plotting and statistical analysis were carried out using GraphPad Prism 8.0.1. software. For evaluating significant differences, one-way ANOVA with Tukey’s multiple comparisons post hoc test was used (*p* < 0.05).

## 3. Results and Discussion

### 3.1. Preparation and Solid-State Characterization of Cyclodextrin Complexes 

As the employed complex preparation method has a crucial role in the structural features and performance of the cyclodextrin product obtained, the efficiency of co-grinding, as a solid-state-based approach, was compared to that of the co-evaporation, a solution-based method. DSC and XRPD were used to characterize the co-evaporated and the co-ground products. It was previously postulated that the combined use of the complementary analytical techniques is a powerful tool to examine drug/CD interactions in the solid state, to select the most effective preparation technique, and to optimize the processing conditions of the complex preparation. The interactions in the co-evaporated and co-ground products were further analyzed by ATR–FTIR [11].

**Figure 2:**
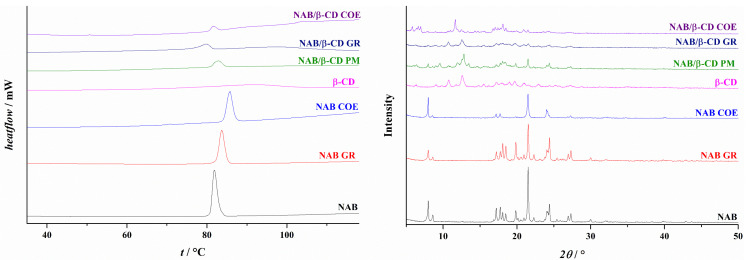
DSC thermograms (**left**) and XRPD diffractograms (**right**) of starting compounds (NAB and β-CD), ground and co-evaporated drug (NAB GR and NAB COE), physical mixture (NAB/β-CD PM), and complexes obtained by co-grinding (NAB/β-CD GR) and co-evaporation (NAB/β-CD COE). The results for the systems prepared with HP-β-CD and SBE-β-CD are presented in Supporting Information (Figures S1 and S2, respectively).

The DSC thermogram of NAB presented a sharp exothermic peak with an onset temperature of 80.9 °C and a fusion enthalpy of 139.0 J/g (Figure 2), corresponding to the melting of crystalline polymorphic form I of the drug [26,27]. This result was confirmed by XRPD analysis (Figure 2), which presented a diffractogram with numerous sharp peaks at 2θ values of 7.96, 8.60, 17.18, 17.75, 18.07, 18.48, 19.83, 21.51, 24.40, 26.94, and 27.31°, which are typical for the NAB’s polymorphic form I [27,28]. The grinding of pure NAB did not significantly change its physicochemical properties. Although a slight reduction of drug residual crystallinity was observed (Table S3), the drug remained in the same polymorphic form. Namely, two polymorphic forms are described for NAB [26,27,28,29]. Polymorph II is a metastable form that transforms into the more stable form I upon slight mechanical contact. Form I is characterized by the onset of melting at a temperature of around 80 °C. In comparison, form II is characterized by melting at a temperature of around 65 °C [26,28]. Furthermore, these two forms can also be distinguished based on XRPD diffractograms, whereby form II is characterized by typical peaks at 6.50, 9.77, 13.04, and 19.61°, which were not observed in the diffractograms of ground NAB (Figure 2). During the grinding, the mechanical energy may be transferred to the treated material by impact or shearing, depending on the number and diameter of the grinding balls. In general, when milling is performed using a large number of smaller balls, as was the case in our experiments, the main mechanism of the energy transfer is the shearing. The transferred energy first reduces particle size to a critical threshold. Further energy input causes the amorphization of the treated material, increasing the molecular mobility and reactivity of the sample [30,31]. However, this state is unstable, and the mechanochemically activated system tends to stabilize, which, in the case of NAB, leads to the formation of the most stable polymorphic form I. At the same time, only a small fraction of the sample remains in the amorphous form, as suggested by the *RDC* values presented in Table S3. Furthermore, the co-evaporation procedure also had only a minor impact on the crystallinity of the drug, showing a similar reduction in *RDC* as observed for the ground NAB. The drug retained the most stable crystalline form in this sample, presenting a DSC curve and XRPD diffractogram typical for the polymorphic form I of NAB (Figure 2).

When the grinding was performed in the presence of cyclodextrins, a different degree of drug amorphization was observed, depending on the cyclodextrin type and the applied processing parameters (Table S3). The lowest degree of amorphization was obtained for the co-ground NAB with β-CD, presenting an *RDC* of 67.8%. Furthermore, in the case of NAB complexes with β-CD, co-evaporation appears as a more efficient technique, yielding a product with an *RDC* value of 26.6%. Grinding with HP-β-CD and SBE-β-CD was more efficient in establishing the solid-state interaction among the treated compounds, depending highly on the applied grinding frequency. After 120 min of grinding at 20 Hz, the *RDC* for the NAB/HP-β-CD was 75.1%, while processing for the same period but at 30 Hz resulted in a completely amorphous product, as shown by DSC and XRPD analysis (Figure 2). However, the consistency of such product was changed, and it remained compacted on the walls of the grinding jars, which caused the product to be difficult to collect. Therefore, the grinding time was intentionally reduced to 90 min, giving a powdered product with an *RDC* value of 49.2%. For comparison, the *RDC* in the NAB/HP-β-CD sample prepared by co-evaporation was 96.9%, clearly underlying the superior efficiency of the co-griding process in establishing efficient solid-state interactions among the drug and cyclodextrin used. Similar behavior was observed when grinding was performed with SBE-β-CD: processing at the lower grinding frequency (i.e., 20 Hz) for 120 min yielded a product where *RDC* was 84.3%, while increasing the grinding frequency for the same grinding period resulted in a product with *RDC* of 59.7%. On the contrary, the co-evaporation of NAB with SBE-β-CD resulted in a product with an *RDC* of 66.7% (Table S3). Furthermore, in all products with cyclodextrins, regardless of the preparation method employed, the residual drug fraction did not change the polymorphic form, presenting the most stable form—form I—as evident from DSC and XRPD results (Figure 2).

The starting components, their physical mixtures, and solid complexes prepared by grinding were further analyzed by ATR–FTIR spectroscopy. The ATR spectra are given in Figures S3–S5, while the assignation of vibrational bands is in Tables S4–S6. As can be seen, the ATR spectra of the physical NAB/β-CD mixture corresponded to the sum of the spectra of individual components. However, in the ATR spectra of the NAB/β-CD complex prepared by grinding, the shift of characteristic NAB bands, which were previously assigned by P. Govindasamy and S. Gunasekaran [32], to higher wavenumbers was observed, namely, from 1705 to 1706 cm^–1^ (*ν*(C=O)) and from 1633 and 1228 to 1635 and 1230 cm^–1^ (*ν*(CC) and *τ*(HCOC), respectively). In addition, a decrease in band intensities was also observed, indicating the formation of inclusion complexes in the solid state [11,33].

By inspecting the ATR spectra obtained for complexes with CD derivatives (Figures S4 and S5), the similarity with the above-mentioned system can be easily observed. The spectra of physical mixtures are the superposition of those of pure components, while, in the ATR spectra of complexes, the shift and diminishing of NAB vibrational bands intensities were noticed.

The FTIR analyses of complexes prepared by the co-evaporation method reveals the fact that the preparation of the NAB-HP-β-CD complex by grinding was more efficient (Figures S6–S8).

During the grinding, the solid-state interactions between NAB and the cyclodextrin employed are mediated by an amorphous solid phase, allowing greater molecular mobility and the establishment of inclusion and non-inclusion interactions between the compounds [30]. As parent β-CD presents a very strong crystal lattice due to intramolecular hydrogen bonding among the adjacent glucopyranose units’ C2 and C3 hydrogen groups, its potential for amorphization is significantly reduced. Because of that, solid-state interaction in the NAB/β-CD sample during the grinding is restricted, resulting in a product with a high fraction of *RDC*. A careful observation of Figure 2 reveals that most residual diffraction peaks in the NAB/β-CD GR sample are typical of β-CD. The weaker potential of β-CD to form effective solid-state interactions with the drug during co-grinding has also been observed with other drugs, while amorphous CD derivatives appear more effective [12,34,35,36]. In line with this, a relatively high *RDC* observed in the case of NAB/SBE-β-CD was somewhat surprising (Table S3). However, even if the product contains a significant fraction of residual crystalline drug, it could be readily converted into an inclusion complex upon dissolution in water, as demonstrated by Jablan et al. [35].

### 3.2. Solubility and In Vitro Dissolution Properties of the Co-Ground Complexes

**Figure 3:**
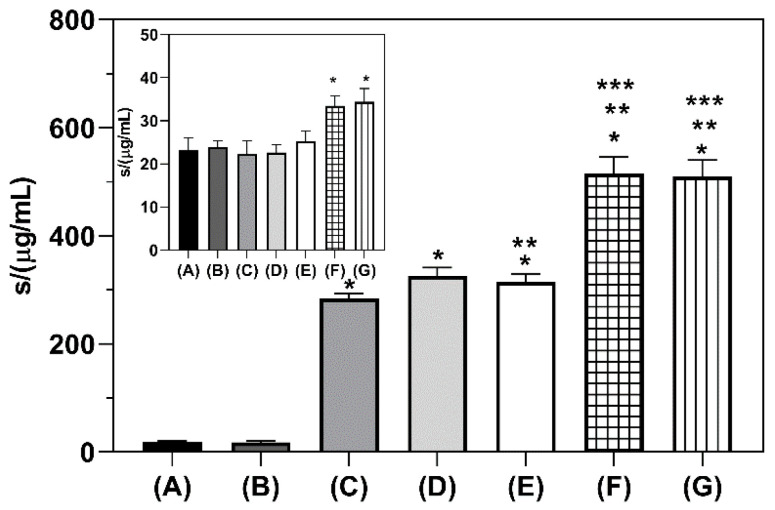
Saturation solubility of NAB and co-ground CD complexes in a hydrochloric acid medium pH 1.2 and a hydrochloric acid medium pH 1.2 with 2% (*w*/*V*) sodium lauryl sulfate (insert) at 37 °C. One asterisk (*) denotes a statistically significant difference (*p* < 0.05) compared to pure NAB, two asterisks (**) denote a statistically significant difference (*p* < 0.05) compared to β-CD complex, and three asterisks (***) denote a statistically significant difference (*p* < 0.05) compared to HP-β-CD complex. Sample codes: NAB (A), NAB GR (B), NAB/β-CD GR (C), NAB/HP-β-CD GR 20 Hz/120 min (D), NAB/HP-β-CD GR 30 Hz/90 min (E), NAB/SBE-β-CD GR 20 Hz/120 min (F), and NAB/SBE-β-CD GR 30 Hz/120 min (G).

In vitro dissolution tests are crucial in drug dosage form development, quality control, and regulatory approval [37]. They are widely used to mimic and predict the in vivo performance of oral dosage forms in the gastrointestinal tract [38]. Media selection is critical when designing in vitro dissolution methods. Selected media should simulate human gastrointestinal fluid to provide an in vitro–in vivo correlation, enabling the development of clinically relevant dissolution methods. At the same time, the selected medium should be as simple as possible to avoid possible analytical interferences [39]. The US Pharmacopeia prescribes a 2% aqueous sodium lauryl sulfate (SLS) solution as a dissolution medium for NAB tablets [40]. Such a medium has been successfully applied to test the dissolution of micronized nabumetone prepared by a rapid expansion supercritical solution process [22]. To provide more relevant conditions to those encountered in the stomach, we have opted to modify this procedure using a hydrochloric acid medium prepared according to the prescription in monograph 5.17.1. Recommendation on dissolution testing of the European Pharmacopoeia [21], with or without 2% SLS. To assess the suitability of such media, we first tested the solubility of NAB and prepared co-ground products with cyclodextrins in such media. The obtained results are presented in Figure 3.

**Figure 4:**
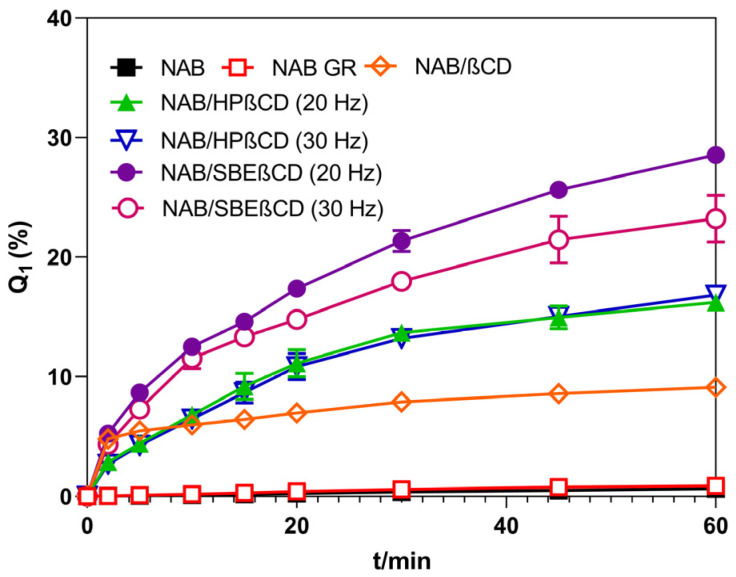
In vitro dissolution profiles of NAB and its co-ground products with CDs in hydrochloric acid medium at 37 °C.

The solubility of tested samples in a hydrochloric acid medium clearly demonstrates the benefits obtained by the cyclodextrin complexation of NAB by grinding, increasing NAB solubility from 14.6 times in the case of β-CD complex up to 26.2 times for SBE-β-CD complexes. These results align with those observed by the phase solubility studies reported in our previous publication [8]. However, when a hydrochloric medium containing 2% (*w*/*V*) SLS was employed, the increase in NAB solubility brought up by cyclodextrin complexation was absent, probably due to the competition of SLS for inclusion complexation with CDs, thus displacing the drug from the complex [41]. In this light, the SLS-containing medium does not seem appropriate for the in vitro dissolution testing of CD complexes of NAB. Furthermore, the addition of an organic solvent like methanol was also considered; however, as organic solvent content in the dissolution medium is restricted to below 5% (*V*/*V*), such an approach did not yield any substantial increase in NAB solubility. Therefore, we have performed the in vitro dissolution studies in non-sink conditions using a hydrochloric acid medium without surfactants, employing conditions reflecting drug dose intake (i.e., 500 mg of NAB) with a glass of water (i.e., 250 mL). The performance of the experiments in such conditions enabled the assessment of cyclodextrin’s performance in the worst-case scenario that may occur during the drug dissolution upon oral administration in vivo. The observed in vitro dissolution profiles of NAB and co-ground CD complexes in such conditions are presented in Figure 4.

The dissolution efficiency (*DE*) parameter, defined as the ratio of the area under the dissolution curve up to a testing time point to the area of the rectangle that describes 100% dissolution up to the same time point, was employed to characterize the obtained dissolution profiles. This enabled the calculation of the dissolution enhancement factor, defined as the ratio between the *DE* of the co-ground sample and the *DE* of the pure drug. The values obtained are presented in Table 1.

To further evaluate the efficiency of co-ground products with CDs in enhancing the dissolution of NAB, the similarity factor *f*_2_ was calculated using a model-independent mathematical method. Several FDA and EMA guidelines adopted the *f*_2_ comparison as a criterion to estimate the similarity between two in vitro dissolution profiles. The two drug products are considered similar when the *f*_2_ value falls between 50 and 100, where an *f*_2_ of 100 signifies that the two profiles are identical, while an average variation of 10% at all determined time points leads to an *f*_2_ value of 50 [24].

Pure NAB’s in vitro dissolution profile is characterized by low dissolution efficiency due to its poor solubility in an aqueous medium (Table 1). The ground NAB sample presented almost the same dissolution profile (*f*_2_ = 99.62 ± 0.23) and only an insignificant increase in dissolution efficiency (*p* > 0.05), in line with the lack of significant drug amorphization obtained by grinding. Co-grinding with CDs significantly increased the dissolution efficiency of the drug, depending on the employed CD derivative and applied grinding conditions. However, in the case of the NAB/β-CD sample, the observed dissolution enhancement did not lead to a substantially different dissolution profile, presenting an *f*_2_ of 58.32 ± 1.15 compared to pure NAB. Only grinding with HP-β-CD and SBE-β-CD yielded products that showed dissolution profiles that were substantially different from that of pure NAB (*f*_2_ values were 48.61 ± 0.76, 48.74 ± 0.16, 37.13 ± 0.05, and 40.87 ± 0.87 for HP-β-CD and SBE-β-CD complexes ground at 20 and 30 Hz, respectively). Interestingly, complexes prepared with the HP-β-CD at different grinding frequencies presented identical in vitro dissolution profiles, irrespective of the different amorphization degrees of the drugs obtained (Table 1, *f*_2_ = 93.90 ± 3.38). In the case of co-ground complexes with SBE-β-CD, an anomalous behavior was observed, where the product prepared by grinding at 20 Hz (characterized by a lower degree of nabumetone amorphization) presented a higher dissolution enhancement index than that obtained at 30 Hz, which contained a higher fraction of the amorphous drug (Table 1). Such unusual behavior may be attributed to more pronounced product compaction, as discussed earlier.

### 3.3. In Vitro Dissolution and Gastrointestinal Permeability Studies in a Low-Volume Environment

An in vitro assessment of dissolution–absorption processes was performed using a device consisting of a low-volume absorption chamber separated from a low-volume donor chamber with a biomimetic gastrointestinal tract permeation membrane. This device is proposed as a valuable tool for understanding the complex interplay between solubility, permeability, and dissolution rate in vitro, enabling the prediction of in vivo outcomes [42].

#### 3.3.1. In Vitro Dissolution Study at pH 6.8

**Figure 5:**
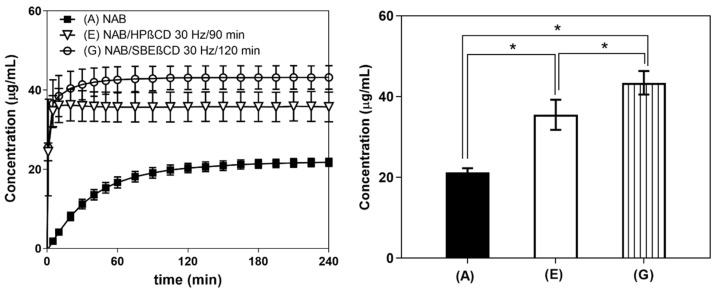
The 4-hour dissolution profiles of NAB (A), NAB/HP-β-CD 30 Hz/90 min (E), and NAB/SBE-β-CD 30 Hz/120 min (G) in PBS, pH 6.8, at 37 °C (**left**) and the thermodynamic solubility of the samples at 24 h (37 °C, PBS pH 6.8) (**right**). Significant differences are indicated with an asterisk: * *p* < 0.05 (n = 3, one-way ANOVA, Tukey’s multiple comparisons test).

As Figure 5 shows, the cyclodextrins-containing nabumetone samples presented a greater dissolution rate than the API alone, as equilibrium was reached within 9 min and 50 min for NAB+HP-β-CD and NAB+SBE-β-CD, respectively. Meanwhile, NAB reached equilibrium only after 1.5 h. The equilibrium concentrations at 24 h were the following: 21.02 µg/mL for NAB, 35.53 µg/mL for NAB+HP-β-CD, and 43.41 µg/mL for NAB+SBE-β-CD. The different solubilities with respect to that observed in the hydrochloric medium may be attributed to a different ionic composition of the media [43]. Regardless, SBE-β-CD was shown to be the most effective solubilizing agent, allowing the highest concentration of nabumetone in the aqueous medium.

#### 3.3.2. In Vitro Gastrointestinal Flux and Permeability Measurements

**Figure 6:**
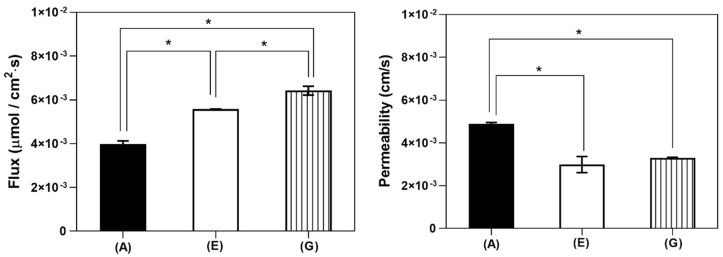
In vitro gastrointestinal flux (**left**) and permeability (**right**) of NAB (A), NAB/HP-β-CD 30 Hz/90 min (E), and NAB/SBE-β-CD 30 Hz/120 min (G). Significant differences are indicated with an asterisk: * *p* < 0.05 (n = 3, one-way ANOVA, Tukey’s multiple comparisons test).

The fluxes of dissolved NAB across the biomimetic membrane are presented in Figure 6, clearly presenting the enhanced mass transport observed in the case of co-ground CD products. The higher solubility of NAB/HP-β-CD and NAB/SBE-β-CD created a higher local concentration of the drug available for the permeation, thus increasing the drug thermodynamic activity at the membrane, resulting in higher flux values [44]. This is significant, as it may enable faster achievement and higher levels of maximum drug plasma concentration, thus leading to a faster onset of drug action, as observed in the case of the CD complexes of piroxicam [45]. However, the apparent permeability coefficients for co-ground products with HP-β-CD and SBE-β-CD appeared lower than that of the pure drug. It should be noted that this result is only a consequence of higher drug concentrations in the donor compartment, as observed by in vitro dissolution studies (Figure 5). In line with this, the observed flux values appear more relevant in predicting the effect of CDs on the intestinal absorption of the drug from the tested formulations.

### 3.4. Effect of Cyclodextrins on NAB Chemical and Photochemical Stability

The stability of active pharmaceutical ingredients and their formulations is a critical parameter that ensures their safety and efficacy. Previously, it was shown that β-CDs can influence the chemical stability of a drug by increasing or even decreasing its stability [4,9,46]. Therefore, in the continuation of our study, based on the in vitro dissolution results, the stability of pure and co-ground NAB, CDs, and NAB complexes with HP-β-CD and SBE-β-CD was investigated. Before analysis, to be able to characterize and identify NAB degradation products [47], the stability-indication UHPLC–DAD–MS method for the quantitative determination of NAB in the presence of its degradation products was developed and validated (Tables S1 and S2). For that purpose, stress tests were performed in hydrolytic (acidic, neutral, and basic) and oxidative conditions both at room temperature and 80 °C.

At first, the analysis of pure NAB was performed. The total ion current chromatogram (TIC) of NAB and its MS spectrum are shown in Figures S9 and S10. The base peak (*m/z* 171) corresponds to the NAB in-source fragment ion, previously described by Valero and Costa [48] and by Wolff et al. [49], while peaks at *m/z* 229 and 251 to [NAB+H]^+^ and [NAB+Na]^+^ ions, respectively. All data are given in Table S7.

**Figure 7:**
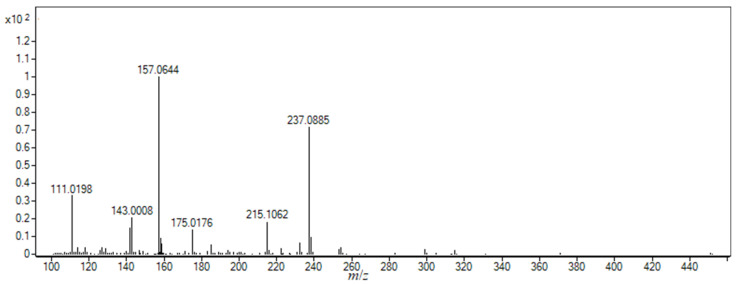
HRMS spectrum of degradation product DP 1 (*t*_R_ = 10.9 min) formed during the acidic hydrolytic degradation.

Nabumetone was prone to hydrolytic degradation only in acidic conditions, both alone and in systems with HP-β-CD and SBE-β-CD (Figure S11), yielding one degradation product (DP 1), which was noticed at the retention time of 10.9 min. The observed accurate *m*/*z* value of the singly protonated ion of DP 1 was 215.1062 (Figure 7). Structural characterization was based on the results of HRMS and MS/MS analysis (Table S8). This compound, 4-(6-hydroxy-2-naphthtyl)-2-butanone, was previously recognized as one of the pharmacologically inactive NAB metabolites [50,51]. 

The hydroxypropylated derivative of β-CD was also prone to hydrolytic degradation, yielding various differently substituted isomeric hydroxypropylated linear maltooligomers (Figure S12). The exact identification of degradation products is technically impossible, as HP-β-CD is a mixture of hundreds of thousands of isomeric species [52]. No degradation products were observed under neutral and basic hydrolytic conditions, which is in accordance with literature data on β-CD stability in neutral and basic media [53].

Nabumetone was not susceptible to oxidative degradation both at room temperature and at elevated temperatures. However, the presence of HP-β-CD and SBE-β-CD in their co-ground samples led to the formation of several degradation products only at elevated temperatures. The total ion chromatograms are given in Figure S13. Ten degradation products were identified in total as a result of the extensive HRMS and MS/MS analyses of protonated molecular ions (Tables S9–S18). The hydroxylation of the naphthalene moiety of NAB was the most pronounced degradation process. Such extensive hydroxylation can only be induced by hydroxy radicals. Indeed, hydrogen peroxide contains a very weak O–O bond, which can easily cleave to form hydroxyl radicals (^•^OH), a reactive oxidative reagent [54]. Likely, the presence of HP-β-CD and SBE-β-CD promote the ^•^OH formation, making the hydroxylation of naphthalene moiety the dominating process at elevated temperature.

**Figure 8:**
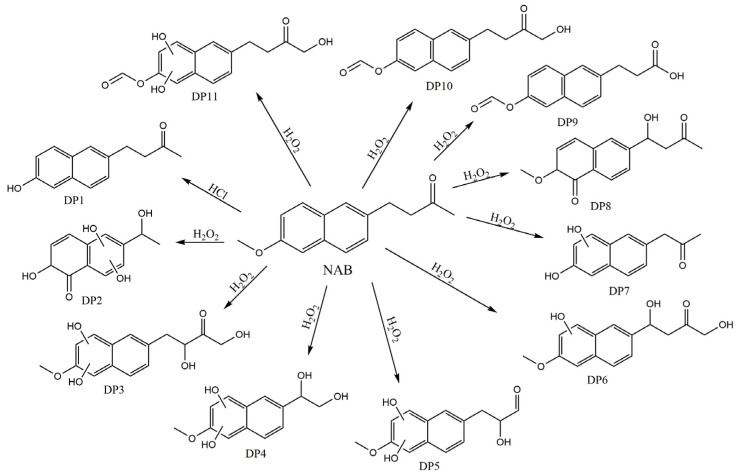
Proposed degradation products of NAB in hydrolytic (HCl) and oxidative conditions (H_2_O_2_). Experimental conditions: 2M HCl; 3% H_2_O_2_ at 80 °C.

The proposed structures of NAB degradation products obtained during forced degradation studies are shown in Figure 8. It should be emphasized that the structures are proposed based on HRMS spectra and MS/MS data. The unambiguous determination of the structures will be confirmed by the isolation of degradation products and their analysis by NMR spectroscopy, which is the subject of upcoming investigations.

Nabumetone, both alone and in systems with HP-β-CD and SBE-β-CD, was not prone to photodegradation. In all cases, the NAB content in samples subjected to photostability studies was within the specification of 98.5–102.6 of the control samples (Figure S14). To obtain deeper knowledge about possible minor degradation products formed during these studies, we performed a UHPLC–HRMS analysis. No degradation products were observed in any of the samples. The total ion chromatograms are given in the supporting information (Figure S15).

All co-ground systems showed satisfying chemical stability during the long-term stability studies. Both pure NAB and co-ground NAB were not prone to degradation. The recovered NAB content at the end of the 9-month testing was 93.5–100.5 of the initial content in all cases (Figure S16). No degradation products were observed in any of the samples during the UHPLC–HRMS analysis as well. The total ion chromatograms are given in the supporting information (Figure S17).

## 4. Conclusions

Grinding in high-energy vibrational mills is a sustainable, solvent-free, and eco-friendly approach for developing CD complexes of poorly soluble drugs like NAB. Processing NAB with CDs yielded powdered products, where the level of the drug amorphization depended on the CD derivative used and the grinding conditions applied. This rapid, one-step technological approach enables the easy control of the process by optimizing grinding time and frequency. Co-ground complexes with SBE-β-CD and HP-β-CD showed the most pronounced in vitro drug dissolution enhancement in both gastric and intestinal media. Enhanced dissolution has led to increased drug flux across the biomimetic membranes, indicating the possibility of achieving a faster onset of drug action upon oral administration of such co-ground complexes, which still needs to be verified in vivo.

Although forced degradation studies revealed various degradants originating from the drug and the CDs used, the co-ground NAB complexes with SBE-β-CD and HP-β-CD showed acceptable photo- and long-term stability, indicating their suitability for further pharmaceutical product development.

## Figures and Tables

**Table 1:** Processing parameters, dissolution efficiency, and dissolution enhancement factor for the prepared co-ground samples.

Sample	Cyclodextrin	Grinding Frequency (Hz)	Grinding Time (min)	Dissolution Efficiency (%)	Dissolution Enhancement Factor
NAB	-	-	-	0.17 ± 0.03	-
NAB GR	-	20	120	0.26 ± 0.03	1.53 ± 0.19
NAB/β-CD GR	β-CD	20	120	3.70 ± 0.23	21.94 ± 1.35
NAB/HP-β-CD GR 20 Hz	HP-β-CD	20	120	5.87 ± 0.28	34.86 ± 1.64
NAB/HP-β-CD GR 30 Hz	HP-β-CD	30	90	5.81 ± 0.04	34.49 ± 0.26
NAB/SBE-β-CD GR 20 Hz	SBE-β-CD	20	120	9.83 ± 0.05	58.30 ± 0.28
NAB/SBE-β-CD GR 30 Hz	SBE-β-CD	30	120	8.30 ± 0.41	49.25 ± 2.45

## Data Availability

The original contributions presented in the study are included in the article/Supplementary Material; further inquiries can be directed to the corresponding author.

## Supplementary Materials

The following supporting information can be downloaded at: https://www.mdpi.com/article/10.3390/pharmaceutics16121493/s1, The SI material published online alongside the article concerns the details for UHPLC and MS analyses (Tables S1 and S2); characterization of NAB complexes in the solid state by DSC and PXRD (Figures S1 and S2, Table S3); ATR–FTIR analysis of the complexes (Figures S3–S8 and Tables S4–S6); chemical stability (Figures S9–S13 and Tables S7–S18); photochemical stability (Figure S14 and S15); and long-term stability (Figures S16 and S17).

## References

[1] Jansook P., Ogawa N., Loftsson T. (2018). Cyclodextrins: Structure, Physicochemical Properties and Pharmaceutical Applications. Int. J. Pharm..

[2] Kali G., Haddadzadegan S., Bernkop-Schnürch A. (2024). Cyclodextrins and Derivatives in Drug Delivery: New Developments, Relevant Clinical Trials, and Advanced Products. Carbohydr. Polym..

[3] Riccio V.B.F., Meneguin A.B., Baveloni F.G., de Antoni A.J., Robusti L.M.G., Gremião M.P.D., Ferrari P.C., Chorilli M. (2023). Biopharmaceutical and Nanotoxicological Aspects of Cyclodextrins for Non-Invasive Topical Treatments: A Critical Review. J. Appl. Toxicol..

[4] Aiassa V., Garnero C., Zoppi A., Longhi M.R. (2023). Cyclodextrins and Their Derivatives as Drug Stability Modifiers. Pharmaceuticals.

[5] Kovacs T., Nagy P., Panyi G., Szente L., Varga Z., Zakany F. (2022). Cyclodextrins: Only Pharmaceutical Excipients or Full-Fledged Drug Candidates?. Pharmaceutics.

[6] Ferreira L., Campos J., Veiga F., Cardoso C., Paiva-Santos A.C. (2022). Cyclodextrin-Based Delivery Systems in Parenteral Formulations: A Critical Update Review. Eur. J. Pharm. Biopharm..

[7] Jacob S., Nair A.B. (2018). Cyclodextrin Complexes: Perspective from Drug Delivery and Formulation. Drug Dev. Res..

[8] Klarić D., Kelrajter M., Čikoš A., Budimir A., Galić N. (2024). Inclusion Complexes of Nabumetone with β-Cyclodextrins: Spectroscopic, Spectrometric and Calorimetric Studies in Solution. J. Mol. Liq..

[9] Špehar T.K., Pocrnić M., Klarić D., Bertoša B., Čikoš A., Jug M., Padovan J., Dragojević S., Galić N. (2021). Investigation of Praziquantel/Cyclodextrin Inclusion Complexation by NMR and LC-HRMS/MS: Mechanism, Solubility, Chemical Stability, and Degradation Products. Mol. Pharm..

[10] Pocrnić M., Hoelm M., Ignaczak A., Čikoš A., Budimir A., Tomišić V., Galić N. (2024). Inclusion Complexes of Loratadine with β-Cyclodextrin and Its Derivatives in Solution. Integrated Spectroscopic, Thermodynamic and Computational Studies. J. Mol. Liq..

[11] Mura P. (2015). Analytical Techniques for Characterization of Cyclodextrin Complexes in the Solid State: A Review. J. Pharm. Biomed. Anal..

[12] Jug M., Mura P.A. (2018). Grinding as Solvent-Free Green Chemistry Approach for Cyclodextrin Inclusion Complex Preparation in the Solid State. Pharmaceutics.

[13] Nicoletti C.D., de Sá Haddad Queiroz M., de Souza Lima C.G., de Carvalho da Silva F., Futuro D.O., Ferreira V.F. (2020). An Improved Method for the Preparation of β-Lapachone:2-Hydroxypropyl-β-Cyclodextrin Inclusion Complexes. J. Drug Deliv. Sci. Technol..

[14] Cabrera-Quiñones N.C., López-Méndez L.J., Guadarrama P. (2023). Inclusion and Non-Inclusion Complexes between Curcumin and β-Cyclodextrin with High-Curcumin Loading and Enhanced Aqueous Solubility Obtained by Mechanochemistry. ChemistrySelect.

[15] Bannwarth B. (2008). Safety of the Nonselective NSAID Nabumetone: Focus on Gastrointestinal Tolerability. Drug Saf..

[16] Hedner T., Samulesson O., Währborg P., Wadenvik H., Ung K.A., Ekbom A. (2004). Nabumetone: Therapeutic Use and Safety Profile in the Management of Osteoarthritis and Rheumatoid Arthritis. Drugs.

[17] Bensouilah N., Boutemeur-Kheddis B., Bensouilah H., Meddour I., Abdaoui M. (2017). Host-Guest Complex of Nabumetone: β-Cyclodextrin: Quantum Chemical Study and QTAIM Analysis. J. Incl. Phenom. Macrocycl. Chem..

[18] Al-Rawashdeh N.A.F. (2005). Interactions of Nabumetone with γ-Cyclodextrin Studied by Fluorescence Measurements. J. Incl. Phenom. Macrocycl. Chem..

[19] Valero M., Tejedor J., Rodríguez L.J. (2007). Encapsulation of Nabumetone by Means of Drug: (β-Cyclodextrin)2:Polyvinylpyrrolidone Ternary Complex Formation. J. Lumin..

[20] Goyenechea N., Sánchez M., Vélaz I., Martín C., Martínez-Ohárriz C., Zornoza A. (2002). Interactions of Nabumetone with Cyclodextrins in Solution and in the Solid State. J. Incl. Phenom. Macrocycl. Chem..

[21] (2024). 5.17.1. Recommendations on Dissolution Testing. European Pharmacopeia.

[22] Su C.S., Tang M., Chen Y.P. (2009). Micronization of Nabumetone Using the Rapid Expansion of Supercritical Solution (RESS) Process. J. Supercrit. Fluids.

[23] Anderson N.H., Bauer M., Boussac N., Khan-Malek R., Munden P., Sardaro M. (1998). An Evaluation of Fit Factors and Dissolution Efficiency for the Comparison of in Vitro Dissolution Profiles. J. Pharm. Biomed. Anal..

[24] Xie F., Ji S., Cheng Z. (2015). In Vitro Dissolution Similarity Factor (F2) and in Vivo Bioequivalence Criteria, How and When Do They Match? Using a BCS Class II Drug as a Simulation Example. Eur. J. Pharm. Sci..

[25] ICH Q1B, Stability Testing: Photostability Testing of New Drug Substances and Products. https://www.ema.europa.eu/en/documents/scientific-guideline/ich-q-1-b-photostability-testing-new-active-substances-and-medicinal-products-step-5_en.pdf.

[26] Price C.P., Grzesiak A.L., Lang M., Matzger A.J. (2002). Polymorphism of Nabumetone. Cryst. Growth Des..

[27] Türk M., Bolten D. (2016). Polymorphic Properties of Micronized Mefenamic Acid, Nabumetone, Paracetamol and Tolbutamide Produced by Rapid Expansion of Supercritical Solutions (RESS). J. Supercrit. Fluids.

[28] Chyall L.J., Tower J.M., Coates D.A., Houston T.L., Childs S.L. (2002). Polymorph Generation in Capillary Spaces: The Preparation and Structural Analysis of a Metastable Polymorph of Nabumetone. Cryst. Growth Des..

[29] Suresh K., Ashe J.S., Matzger A.J. (2019). Far-Infrared Spectroscopy as a Probe for Polymorph Discrimination. J. Pharm. Sci..

[30] Tan D., Loots L., Friščić T. (2016). Towards Medicinal Mechanochemistry: Evolution of Milling from Pharmaceutical Solid Form Screening to the Synthesis of Active Pharmaceutical Ingredients (APIs). Chem. Commun..

[31] Julien P.A., Friščić T. (2022). Methods for Monitoring Milling Reactions and Mechanistic Studies of Mechanochemistry: A Primer. Cryst. Growth Des..

[32] Govindasamy P., Gunasekaran S. (2015). Experimental and Theoretical Studies of (FT-IR, FT-Raman, UV–Visible and DFT) 4-(6-Methoxynaphthalen-2-Yl) Butan-2-One. Spectrochim. Acta A Mol. Biomol. Spectrosc..

[33] Cannavà C., Crupi V., Ficarra P., Guardo M., Majolino D., Stancanelli R., Venuti V. (2008). Physicochemical Characterization of Coumestrol/β-Cyclodextrins Inclusion Complexes by UV–Vis and FTIR-ATR Spectroscopies. Vib. Spectrosc..

[34] Mennini N., Bragagni M., Maestrelli F., Mura P. (2014). Physico-Chemical Characterization in Solution and in the Solid State of Clonazepam Complexes with Native and Chemically-Modified Cyclodextrins. J. Pharm. Biomed. Anal..

[35] Jablan J., Bačić I., Kujundžić N., Jug M. (2013). Zaleplon Co-Ground Complexes with Natural and Polymeric β-Cyclodextrin. J. Incl. Phenom. Macrocycl. Chem..

[36] Cugovčan M., Jablan J., Lovrić J., Cinčić D., Galić N., Jug M. (2017). Biopharmaceutical Characterization of Praziquantel Cocrystals and Cyclodextrin Complexes Prepared by Grinding. J. Pharm. Biomed. Anal..

[37] Jug M., Hafner A., Lovrić J., Kregar M.L., Pepić I., Vanić Ž., Cetina-Čižmek B., Filipović-Grčić J. (2018). An Overview of in Vitro Dissolution/Release Methods for Novel Mucosal Drug Delivery Systems. J. Pharm. Biomed. Anal..

[38] Lex T.R., Rodriguez J.D., Zhang L., Jiang W., Gao Z. (2022). Development of In Vitro Dissolution Testing Methods to Simulate Fed Conditions for Immediate Release Solid Oral Dosage Forms. AAPS J..

[39] Grignard E., Taylor R., McAllister M., Box K., Fotaki N. (2017). Considerations for the Development of in Vitro Dissolution Tests to Reduce or Replace Preclinical Oral Absorption Studies. Eur. J. Pharm. Sci..

[40] USP (2024). Monographs, Nabumetone Tablets. United States Pharmacopeia.

[41] Hao L.S., Wang H.X., Wang Y.S., Meng Y.Q., Nan Y.Q. (2023). Inclusion Complexation of Surfactant with β-Cyclodextrin and Its Effect on the Mixed Micellization of Cationic/Anionic Surfactants. Colloids. Surf. A Physicochem. Eng. Asp..

[42] Kádár S., Kennedy A., Lee S., Ruiz R., Farkas A., Tőzsér P., Csicsák D., Tóth G., Sinkó B., Borbás E. (2024). Bioequivalence Prediction with Small-Scale Biphasic Dissolution and Simultaneous Dissolution-Permeation Apparatus—An Aripiprazole Case Study. Eur. J. Pharm. Sci..

[43] Karkossa F., Klein S. (2017). Assessing the Influence of Media Composition and Ionic Strength on Drug Release from Commercial Immediate-Release and Enteric-Coated Aspirin Tablets. J. Pharm. Pharmacol..

[44] Sripetch S., Prajapati M., Loftsson T. (2022). Cyclodextrins and Drug Membrane Permeation: Thermodynamic Considerations. J. Pharm. Sci..

[45] Scarpignato C. (2013). Piroxicam-ß-Cyclodextrin: A GI Safer Piroxicam. Curr. Med. Chem..

[46] Popielec A., Loftsson T. (2017). Effects of Cyclodextrins on the Chemical Stability of Drugs. Int. J. Pharm..

[47] Narayanam M., Handa T., Sharma P., Jhajra S., Muthe P.K., Dappili P.K., Shah R.P., Singh S. (2014). Critical Practical Aspects in the Application of Liquid Chromatography–Mass Spectrometric Studies for the Characterization of Impurities and Degradation Products. J. Pharm. Biomed. Anal..

[48] Valero M., Costa S.M.B. (2003). Photodegradation of Nabumetone in Aqueous Solutions. J. Photochem. Photobiol. A. Chem..

[49] Wolff J.C., Hawtin P.N., Monté S., Balogh M., Jones T. (2001). The Use of Particle Beam Mass Spectrometry for the Measurement of Impurities in a Nabumetone Drug Substance, Not Easily Amenable to Atmospheric Pressure Ionisation Techniques. Rapid. Commun. Mass. Spectrom..

[50] Nobilis M., Mikušek J., Szotáková B., Jirásko R., Holčapek M., Chamseddin C., Jira T., Kučera R., Kuneš J., Pour M. (2013). Analytical Power of LLE–HPLC–PDA–MS/MS in Drug Metabolism Studies: Identification of New Nabumetone Metabolites. J. Pharm. Biomed. Anal..

[51] Nobilis M., Kopecký J., Květina J., Svoboda Z., Pour M., Kuneš J., Holčapek M., Kolářová L. (2003). Comparative Biotransformation and Disposition Studies of Nabumetone in Humans and Minipigs Using High-Performance Liquid Chromatography with Ultraviolet, Fluorescence and Mass Spectrometric Detection. J. Pharm. Biomed. Anal..

[52] Szente L., Szemán J., Sohajda T. (2016). Analytical Characterization of Cyclodextrins: History, Official Methods and Recommended New Techniques. J. Pharm. Biomed. Anal..

[53] Saokham P., Muankaew C., Jansook P., Loftsson T. (2018). Solubility of Cyclodextrins and Drug/Cyclodextrin Complexes. Molecules.

[54] Gabrić A., Hodnik Ž., Pajk S. (2022). Oxidation of Drugs during Drug Product Development: Problems and Solutions. Pharmaceutics.

