# Supplementary material for: Biopharmaceutical Characterization and Stability of Nabumetone–Cyclodextrins Complexes Prepared by Grinding
Source: Pharmaceutics. 2024 Nov 21;16(12):1493. doi: 10.3390/pharmaceutics16121493 (PMC11679744; doi:10.3390/pharmaceutics16121493)
Supplement: Supplementary file 1 [file pharmaceutics-16-01493-s001.zip › pharmaceutics-3312872-supplementary.pdf]

# Biopharmaceutical Characterization and Stability of Nabumetone–Cyclodextrins Complexes Prepared by Grinding

David Klarić [1], Željka Soldin [1], Anna Vincze [2,3], Rita Szolláth [2,3], György Tibor Balogh [2,3], Mario Jug [4,*] and Nives Galić [1,*]

[1] Department of Chemistry, Faculty of Science, University of Zagreb, Horvatovac 102a, 10 000 Zagreb, Croatia
[2] Department of Pharmaceutical Chemistry, Semmelweis University, Hőgyes Endre u. 9., H-1092 Budapest, Hungary
[3] Center for Pharmacology and Drug Research & Development, Semmelweis University, Üllői u. 26. H-1092 Budapest, Hungary
[4] Department of Pharmaceutical Technology, Faculty of Pharmacy and Biochemistry, University of Zagreb, A. Kovačića 1, 10 000 Zagreb, Croatia

# Supporting Information

## Contents

1. Materials and methods .......... 2
2. Characterization of NAB complexes in solid state .......... 3
   2.1. DSC and XRPD .......... 3
   2.2. FT IR spectroscopy .......... 6
3. Effect of β-cyclodextrins on NAB chemical and photochemical stability .......... 11
   3.1. Hydrolytic degradation .......... 11
   3.2. Photostability study .......... 19
   3.3. Long-term stability study .......... 21

## 1. Materials and methods

**Table S1.** Chromatographic parameters for stability-indicating UHPLC-DAD and UHPLC-HRMS methods.

| Parameter | Developed LC method | | |
|---|---|---|---|
| Column | Agilent ZORBAX RRHD Eclipse Plus C18 | | |
| Mobile phase A ($MF_A$) | 0.1 % (*v*/*v*) formic acid in water | | |
| Mobile phase B ($MF_B$) | 0.1 % (*v*/*v*) formic acid in methanol | | |
| | Time / min | $MF_A$ / % | $MF_B$ / % |
| Gradient elution profile | 0 | 95 | 5 |
| | 15 | 5 | 95 |
| Injection volume (UHPLC-HRMS) | 1 µL | | |
| Injection volume (UHPLC-DAD) | 2 µL | | |
| Mobile phase flow | 0.20 mL/min | | |
| Column temperature | 40 °C | | |
| Detection (DAD) | 231, 254, 260, 266, and 275 nm | | |
| Detection (MS) | ESI+ (100 – 1300 *m*/*z*) | | |

**Table S2.** Instrument parameters for acquiring total ion chromatograms, TICs.

| Parameter | Value |
|---|---|
| Sheath gas temperature | 250 °C |
| Sheath gas flow | 11 L/min |
| Nebulizer pressure | 25 psi |
| Capillary voltage | 2500 V |
| Nozzle voltage | 1000 V |
| Drying gas temperature | 200 °C |
| Drying gas flow | 17 L/min |
| Fragmentor voltage | 100 V |

## 2. Characterization of NAB complexes in solid state

### 2.1. DSC and PXRD

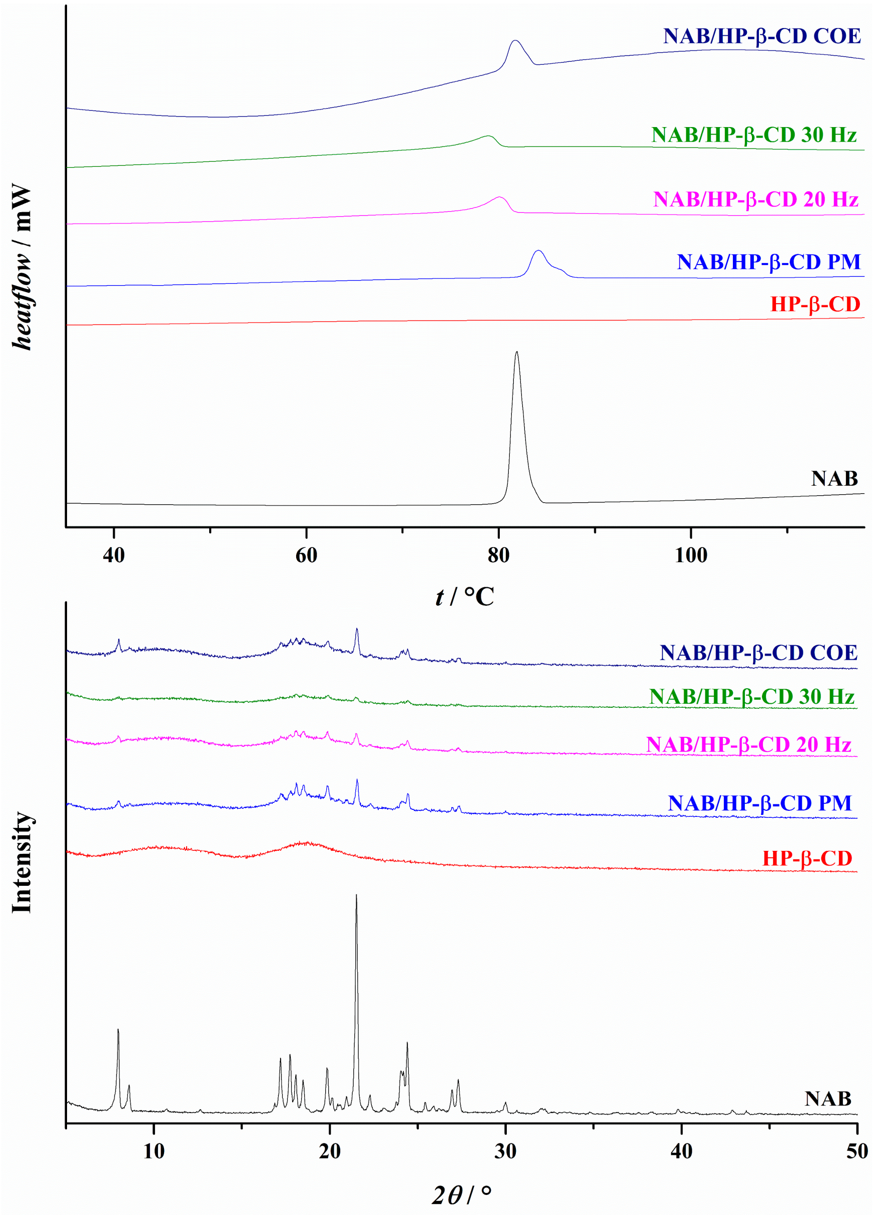


**Figure S1**. DSC thermograms (up) and PXRD diffractograms (down) of starting compounds (NAB and HP-β-CD), ground and co-evaporated drug (NAB GR and NAB COE), physical mixture (NAB/HP-β-CD PM), and complexes obtained by co-grinding (NAB/HP-β-CD GR) and co-evaporation (NAB/HP-β-CD COE).

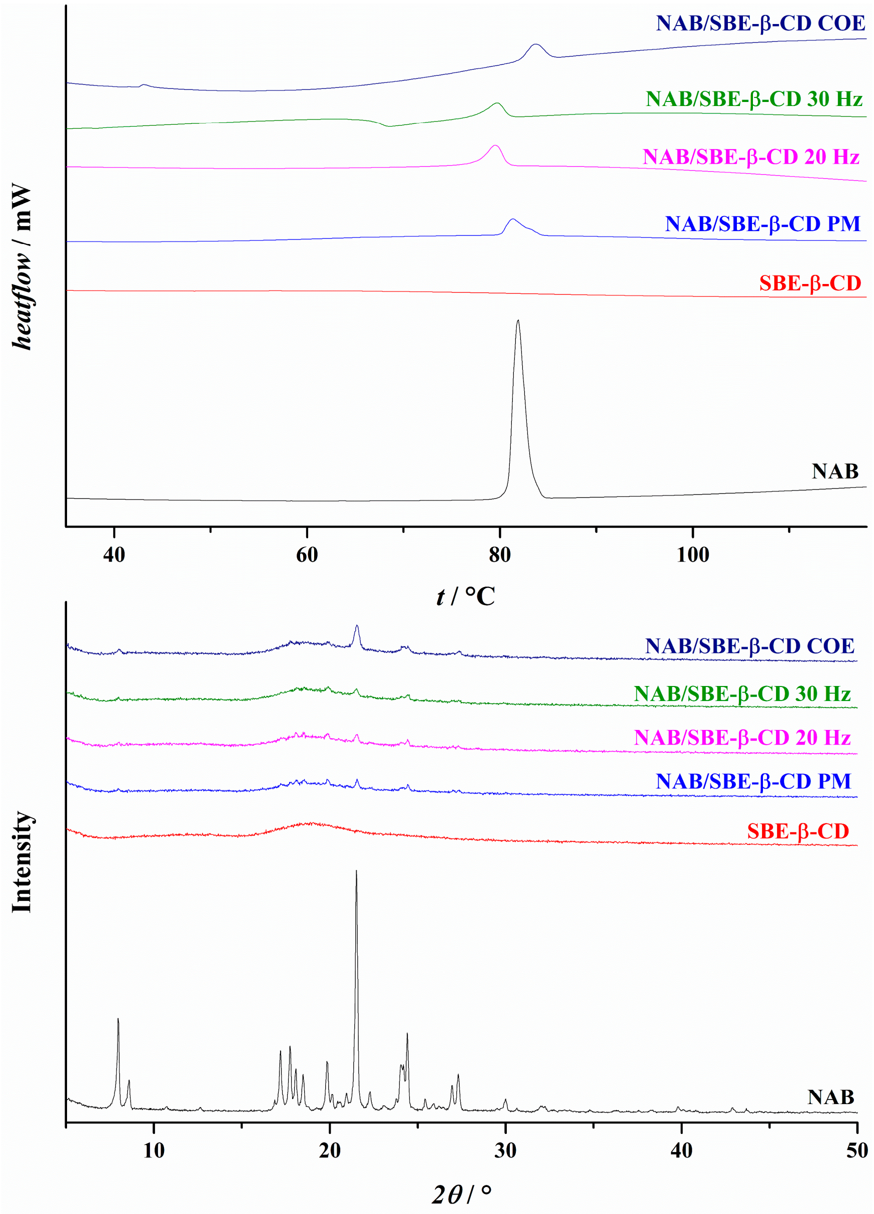


**Figure S2**. DSC thermograms (up) and PXRD diffractograms (down) of starting compounds (NAB and SBE-β-CD), ground and co-evaporated drug (NAB GR and NAB COE), physical mixture (NAB/SBE-β-CD PM), and complexes obtained by co-grinding (NAB/SBE-β-CD GR) and co-evaporation (NAB/SBE-β-CD COE).

**Table S3**. The effect of the processing parameters of the characteristics of the products obtained by co-grinding and co-evaporation: onset temperature ($T_{onset}$), peak temperature ($T_{peak}$), fusion enthalpy ($\Delta H_{fusion}$) and residual drug crystallinity in the product (RDC) obtained by DSC analysis.

| Sample | Grinding time / (min) | Grinding frequency / (Hz) | $T_{onset}$ /(°C) | $T_{peak}$ /(°C) | $\Delta H_{fusion}$ / ($Jg^{-1}$) | RDC / (%) |
|---|---|---|---|---|---|---|
| NAB | 0 | 20 | 80.9 | 81.9 | 139.0 | 100.0 |
| | 30 | | 80.7 | 81.7 | 137.3 | 98.8 |
| | 60 | | 81.8 | 82.9 | 132.6 | 95.4 |
| | 90 | | 80.6 | 81.9 | 141.6 | 101.8 |
| | 120 | | 82.4 | 83.7 | 126.4 | 90.9 |
| | NAB COE | | 84.2 | 85.8 | 131.9 | 95.1 |
| NAB/β-CD | 0 | 20 | 81.6 | 83.4 | 120.5 | 88.5 |
| | 30 | | 79.1 | 80.9 | 111.6 | 82.0 |
| | 60 | | 78.8 | 80.6 | 99.4 | 73.1 |
| | 90 | | 77.9 | 80.4 | 97.7 | 71.8 |
| | 120 | | 76.7 | 79.9 | 92.3 | 67.8 |
| | NAB/β-CD COE | | 80.5 | 81.7 | 36.9 | 26.6 |
| NAB/HP-β-CD | 0 | 20 | 82.7 | 84.1 | 136.3 | 100.2 |
| | 30 | | 79.8 | 81.7 | 123.3 | 90.7 |
| | 60 | | 79.1 | 81.3 | 123.9 | 91.1 |
| | 90 | | 80.2 | 82.7 | 110.6 | 81.3 |
| | 120 | | 77.1 | 80.0 | 102.2 | 75.1 |
| | 0 | 30 | 82.7 | 84.1 | 136.3 | 101.4 |
| | 30 | | 78.1 | 80.2 | 113.2 | 84.2 |
| | 60 | | 77.7 | 80.4 | 91.3 | 67.9 |
| | 90 | | 75.4 | 78.9 | 66.1 | 49.2 |
| | 120 | | - | - | - | 0 |
| | NAB/HP-β-CD COE | | 80.3 | 81.7 | 134.5 | 96.9 |
| NAB/SBE-β-CD | 0 | 20 | 80.0 | 81.2 | 123.1 | 90.4 |
| | 30 | | 79.5 | 81.1 | 131.4 | 96.6 |
| | 60 | | 79.7 | 81.7 | 124.0 | 91.1 |
| | 90 | | 80.4 | 82.6 | 122.6 | 90.1 |
| | 120 | | 77.6 | 79.4 | 114.8 | 84.3 |
| | 0 | 30 | 80.0 | 81.2 | 123.1 | 91.6 |
| | 30 | | 80.0 | 81.8 | 107.4 | 79.9 |
| | 60 | | 78.1 | 80.2 | 106.1 | 78.9 |
| | 90 | | 77.0 | 79.2 | 91.9 | 68.4 |
| | 120 | | 77.5 | 79.7 | 80.2 | 59.7 |
| | NAB/SBE-β-CD COE | | 82.0 | 83.6 | 92.6 | 66.7 |

## 2.2. FT IR spectroscopy

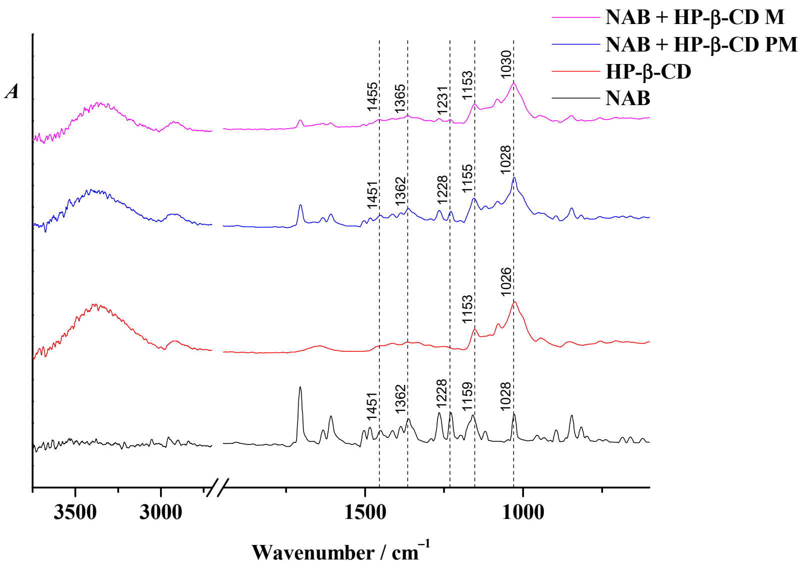


**Figure S3.** FT-IR ATR spectra of NAB (—), β-CD (—), their physical mixture (PM) (—), and complex (M) prepared by grinding at 20 Hz for 120 min in $ZrO_2$ jars (—).

**Table S4.** Assignation of ATR spectra of NAB, β-CD, their physical mixture (PM), and complex (M) prepared by grinding at 20 Hz for 120 min in $ZrO_2$ jars.

| | | ***Wavenumber*** **/ cm$^{-1}$** | | |
|---|---|---|---|---|
| **NAB** | **β-CD** | **NAB+β-CD PM** | **NAB+β-CD M** | **Assignment** |
| - | 3680-3025 | 3691-3025 | 3699-3025 | $\nu$(OH) |
| - | 2930 | 2952 | 2928 | $\nu$(CH) |
| 1706 | - | 1705 | 1706 | $\nu$(C=O) |
| 1608 | - | 1608 | 1609 | $\nu(C{=}C)_{aromat}$ |
| 1485 | - | 1484 | 1484 | $\nu(C{-}C)_{aromat}$ |
| 1451 | - | 1450 | 1447 | $\delta$(HCH)+$\delta$(HCC) |
| 1387 | - | 1388 | 1388 | $\delta$(HCH) |
| 1362 | - | 1363 | 1364 | $\nu$(CC) |
| 1265 | - | 1265 | 1266 | $\delta$(HCC)+$\tau$(HCCC) |
| 1228 | - | 1228 | 1230 | $\nu$(CC)+$\tau$(HCOC) |
| 1159 | - | 1156 | 1154 | $\tau$(HCOC) |
| - | 1153 | 1156 | 1154 | $\nu$(COC)+$\nu$(CC)+$\delta$(COH) |
| 1119 | - | 1119 | 1119 | $\nu$(CC)+$\delta$(HCC) |
| - | 1078 | 1079 | 1079 | $\nu$(CO)+$\nu$(CC)+$\delta$(COH) |
| 1028 | - | 1028 | 1027 | $\nu$(C–O) |
| - | 1027 | 1028 | 1027 | $\nu$(CC)+$\delta$(COH)+$\delta$(CCH)+$\delta$(CCO) |
| 895 | - | 895 | 894 | $\tau$(HCCC) |
| 846 | - | 847 | 847 | $\tau$(HCCC) |
| 816 | - | 815 | 814 | $\nu$(CC) |

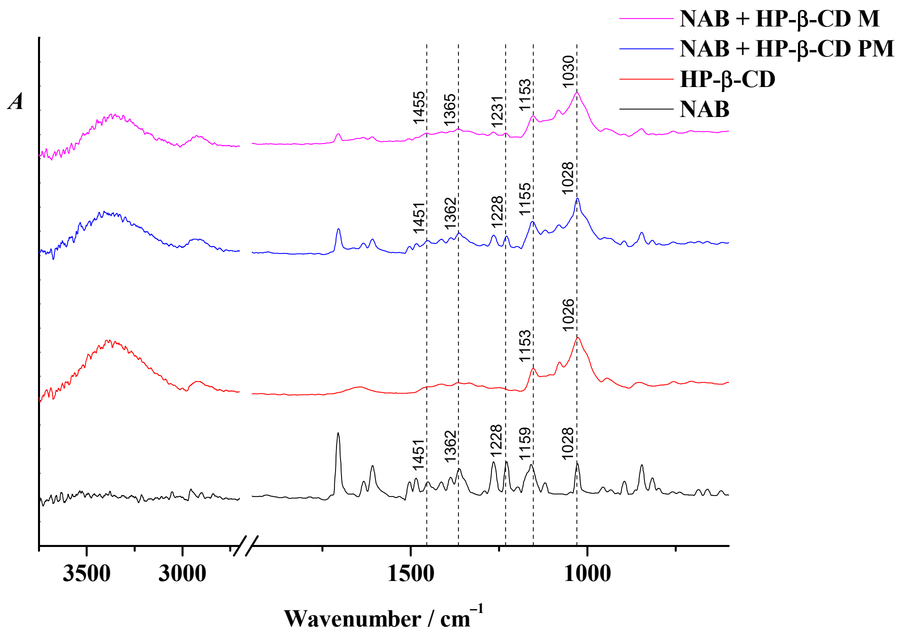


**Figure S4.** FT-IR ATR spectra of NAB (—), HP-β-CD (—), their physical mixture (PM) (—), and complex (M) prepared by grinding at 30 Hz for 90 min in $ZrO_2$ jars (—).

**Table S5.** Assignation of ATR spectra of NAB, HP-β-CD, their physical mixture (PM), and complex (M) prepared by grinding at 30 Hz for 90 min in $ZrO_2$ jars.

| NAB | HP-β-CD | ***Wavenumber* / cm$^{-1}$** NAB+HP-β-CD PM | NAB+HP-β-CD M | Assignment |
|---|---|---|---|---|
| - | 3676-3015 | 3676-3025 | 3683-3046 | *ν*(OH) |
| - | 2929 | 2934 | 2927 | *ν*(CH) |
| 1706 | - | 1705 | 1705 | *ν*(C=O) |
| 1608 | - | 1608 | 1609 | *ν*(C=C)$_{aromat}$ |
| 1485 | - | 1484 | 1481sh | *ν*(C–C)$_{aromat}$ |
| 1451 | - | 1451 | 1455 | *δ*(HCH)+*δ*(HCC) |
| 1387 | - | 1388 | 1388sh | *δ*(HCH) |
| 1362 | - | 1362 | 1365 | *ν*(CC) |
| 1265 | - | 1265 | 1266 | *δ*(HCC)+*τ*(HCCC) |
| 1228 | - | 1228 | 1231 | *ν*(CC)+*τ*(HCOC) |
| 1159 | - | 1155 | 1153 | *τ*(HCOC) |
| - | 1153 | 1155 | 1153 | *ν*(COC)+*ν*(CC)+*δ*(COC) |
| 1119 | - | 1119 | 1119 | *ν*(CC)+*δ*(HCC) |
| - | 1079 | 1081 | 1082 | *ν*(CO)+*ν*(CC)+*δ*(OCH) |
| 1028 | - | 1028 | 1030 | *ν*(C–O) |
| - | 1026 | 1028 | 1030 | *ν*(CC)+ *δ*(OCH)+*δ*(CCH)+*δ*(CCO) |
| 895 | - | 896 | 896 | *τ*(HCCC) |
| 846 | - | 846 | 847 | *τ*(HCCC) |
| 816 | - | 816 | 816 | *ν*(CC) |

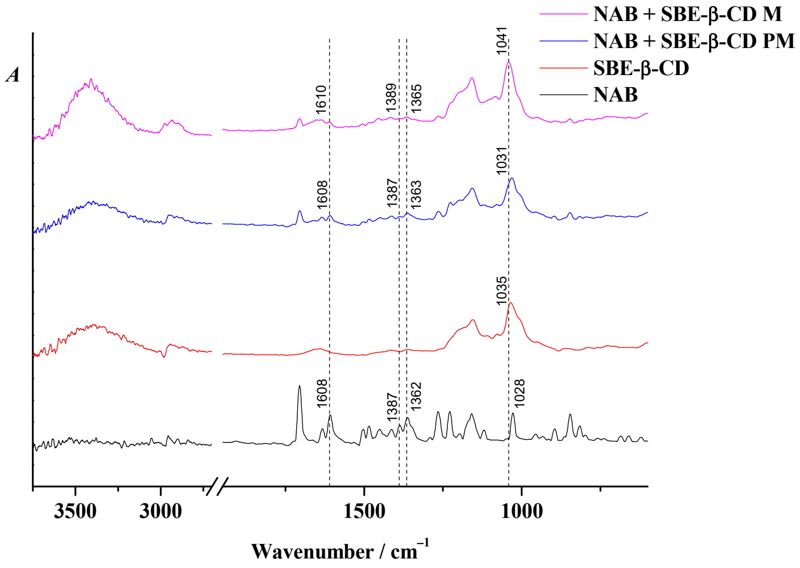


**Figure S5.** FT-IR ATR spectra of NAB (—), SBE-β-CD (—), their physical mixture (PM) (—), and complex (M) prepared by grinding at 30 Hz for 120 min in $ZrO_2$ jars (—).

**Table S6.** Assignation of ATR spectra of NAB, SBE-β-CD, their physical mixture (PM), and complex (M) prepared by grinding at 30 Hz for 90 min in $ZrO_2$ jars.

| NAB | SBE-β-CD | ***Wavenumber* / cm$^{-1}$** NAB+SBE-β-CD PM | NAB+SBE-β-CD M | Assignment |
|---|---|---|---|---|
| - | 3724-2982 | 3709-2987 | 3694-3012 | $\nu$(OH) |
| - | 2943 | 2949 | 2933 | $\nu$(CH) |
| 1706 | - | 1706 | 1705 | $\nu$(C=O) |
| 1608 | - | 1608 | 1610 | $\nu(C{=}C)_{aromat}$ |
| 1485 | - | 1484 | 1484sh | $\nu(C{-}C)_{aromat}$ |
| 1451 | - | 1448 | 1455 | $\delta$(HCH)+$\delta$(HCC) |
| 1387 | - | 1387 | 1389 | $\delta$(HCH) |
| 1362 | - | 1363 | 1365 | $\nu$(CC) |
| 1265 | - | 1265 | 1265 | $\delta$(HCC)+$\tau$(HCCC) |
| 1228 | - | 1227 | 1227sh | $\nu$(CC)+$\tau$(HCOC) |
| 1159 | - | 1157 | 1158 | $\tau$(HCOC) |
| - | 1155 | 1157 | 1158 | $\nu$(COC)+$\nu$(CC)+$\delta$(COC) |
| 1119 | - | - | - | $\nu$(CC)+$\delta$(HCC) |
| - | 1078 | 1079 | 1084 | $\nu$(CO)+$\nu$(CC)+$\delta$(OCH) |
| 1028 | - | 1031 | 1041 | $\nu$(C–O) |
| - | 1035 | 1031 | 1041 | $\nu$(CC)+$\nu$(SO)+$\delta$(OCH)+$\delta$(CCH)+$\delta$(CCO) |
| 895 | - | 897 | 894 | $\tau$(HCCC) |
| 846 | - | 847 | 847 | $\tau$(HCCC) |
| 816 | - | 816 | 814 | $\nu$(CC) |

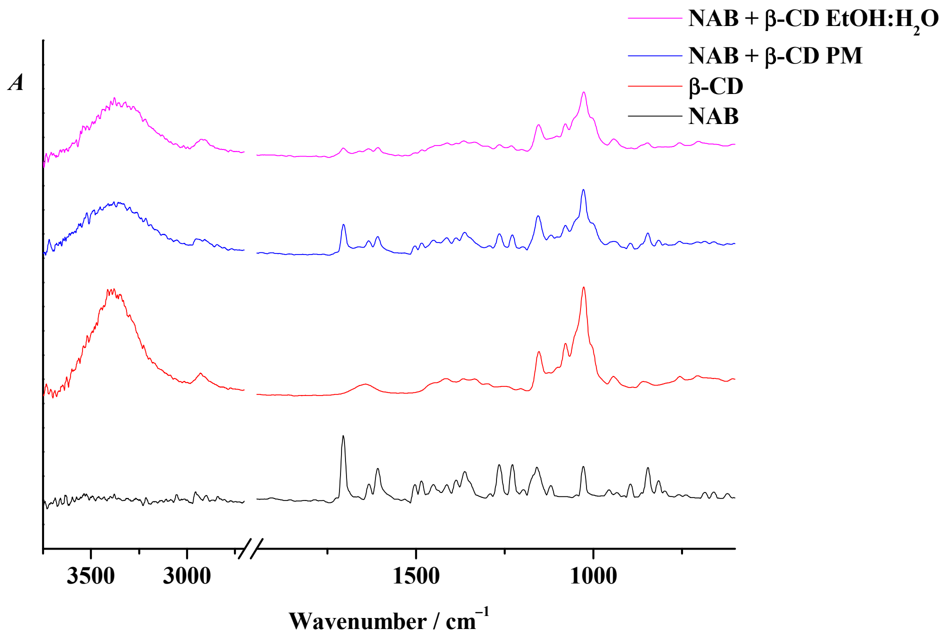


**Figure S6.** FT-IR ATR spectra of NAB (—), β-CD (—), their physical mixture (PM) (—), and complex (M) prepared by co-evaporation from EtOH:$H_2O$ mixture (—).

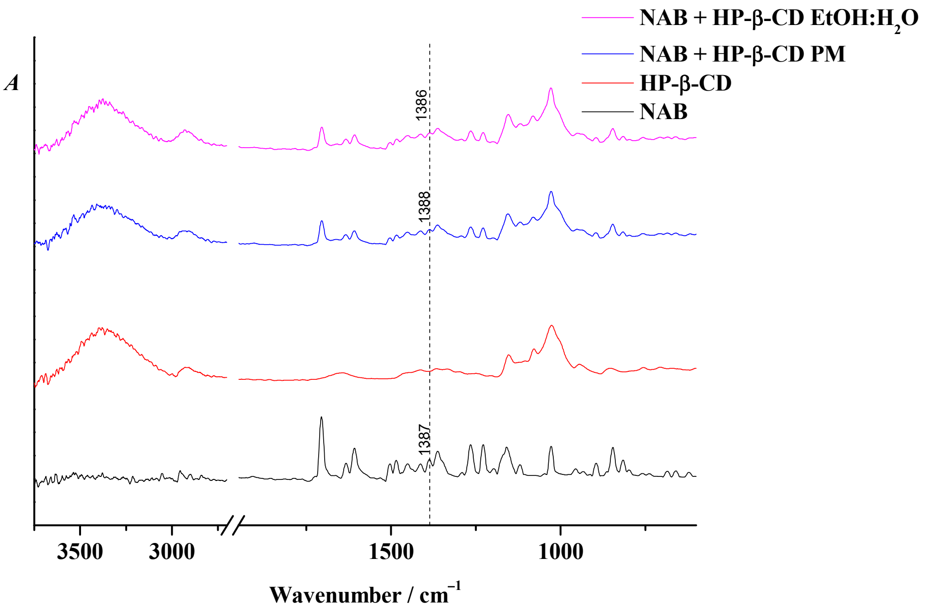


**Figure S7.** FT-IR ATR spectra of NAB (—), HP-β-CD (—), their physical mixture (PM) (—), and complex (M) prepared by co-evaporation from EtOH:$H_2O$ mixture (—).

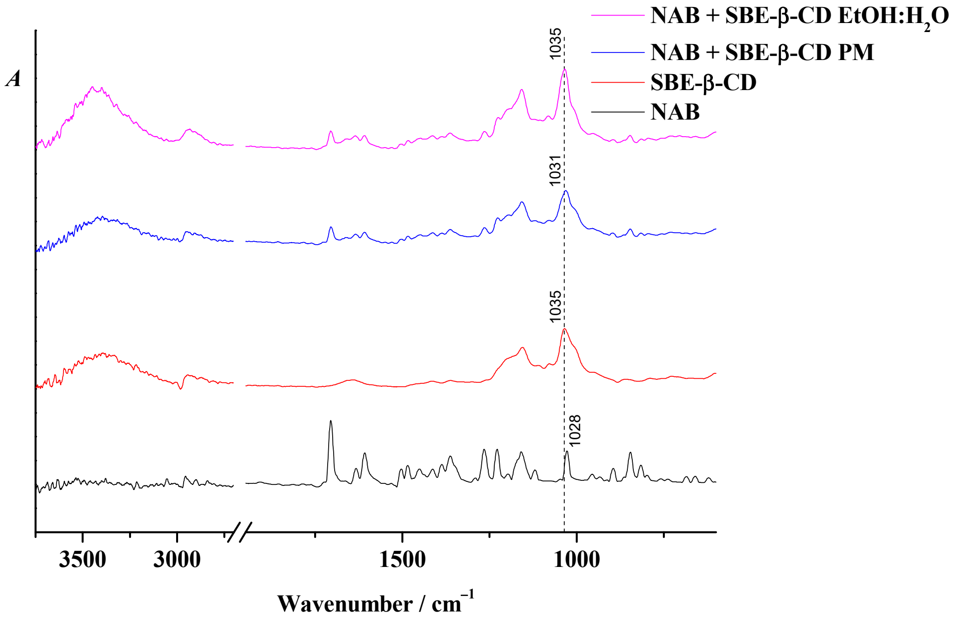


**Figure S8.** FT-IR ATR spectra of NAB (—), SBE-β-CD (—), their physical mixture (PM) (—), and complex (M) prepared by co-evaporation from EtOH:$H_2O$ mixture (—).

## 3. Effect of β-cyclodextrins on NAB chemical and photochemical stability

### 3.1. Hydrolytic degradation

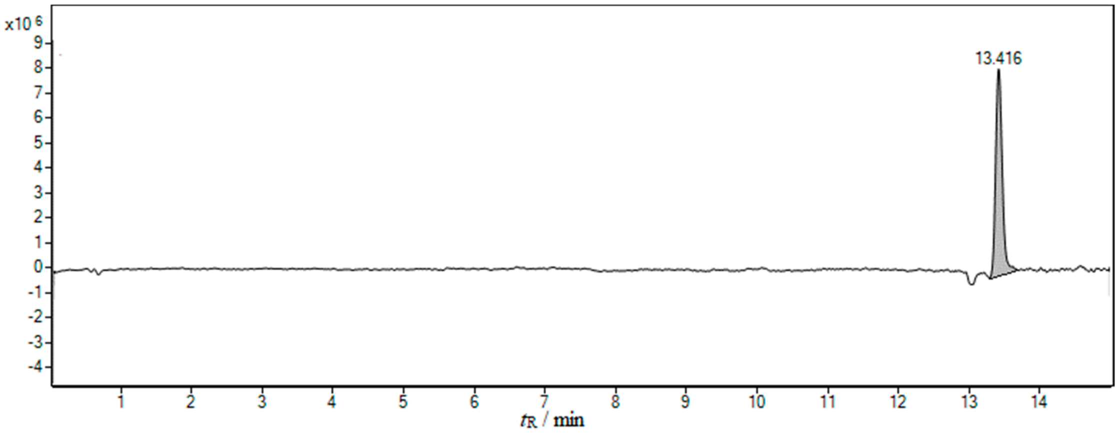


**Figure S9.** Total ion chromatogram of NAB.

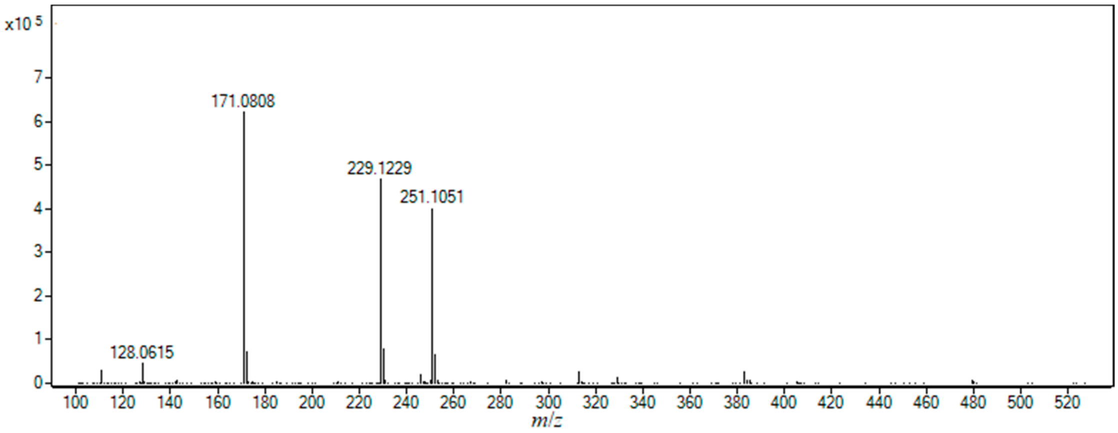


**Figure S10.** MS spectrum of NAB. ($t_R$ = 13.4 min).

**Table S7.** Measured and calculated *m*/*z* values, relative intensities, errors and asignation of signals in MS spectrum of NAB ($t_R$ = 13.4 min).

| Measured *m*/*z* | Calculated *m*/*z* | Error / ppm | Relative intensity / % | Assignation |
|---|---|---|---|---|
| 479.2190 | 479.2193 | 0.63 | 1.86 | $[2M+Na]^+$ |
| 251.1051 | 251.1043 | 3.38 | 64.54 | $[M+Na]^+$ |
| 229.1229 | 229.1223 | 2.59 | 75.46 | $[M+H]^+$ |
| 171.0808 | 171.0804 | 2.10 | 100 | $[M+H–58]^+$ |
| 128.0615 | 128.0621 | -4.31 | 7.69 | $[C_{10}H_8]^{•+}$ |

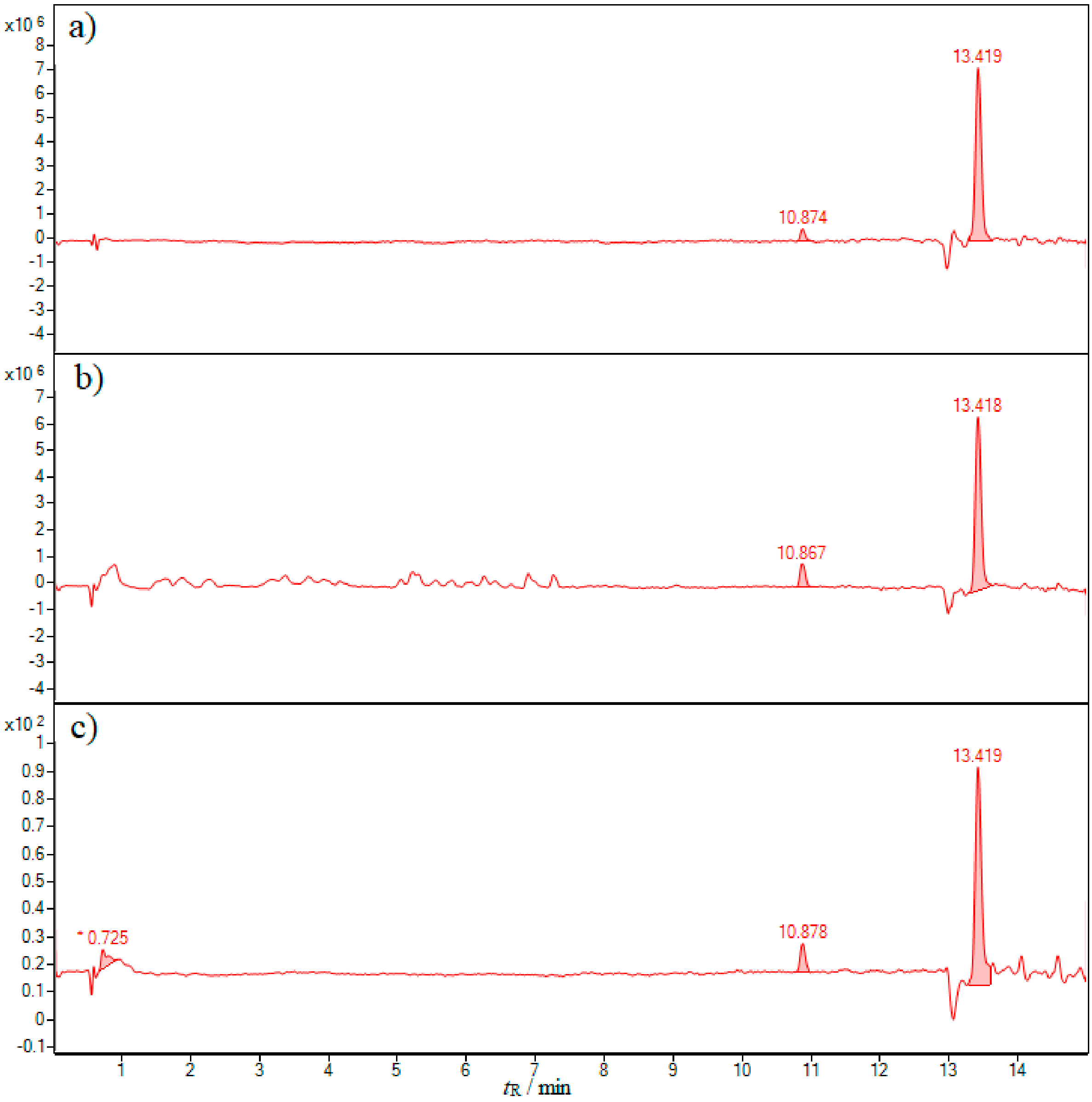


**Figure S11.** Total ion chromatograms of a) NAB, b) NAB-HP-β-CD, and c) NAB-SBE-β-CD under the hydrolytic degradation in acidic media.

**Table S8.** MS/MS spectrum of degradation product DP 1 ($t_R$ = 10.9 min) at collision potential of 10 V.

| **Measured *m*/*z*** | **Rel. intensity** | **Assignation** |
|---|---|---|
| 215,1065 | 3.27 | $[M+H]^+$ |
| 157,0640 | 100 | $[C_{11}H_9O]^+$ |

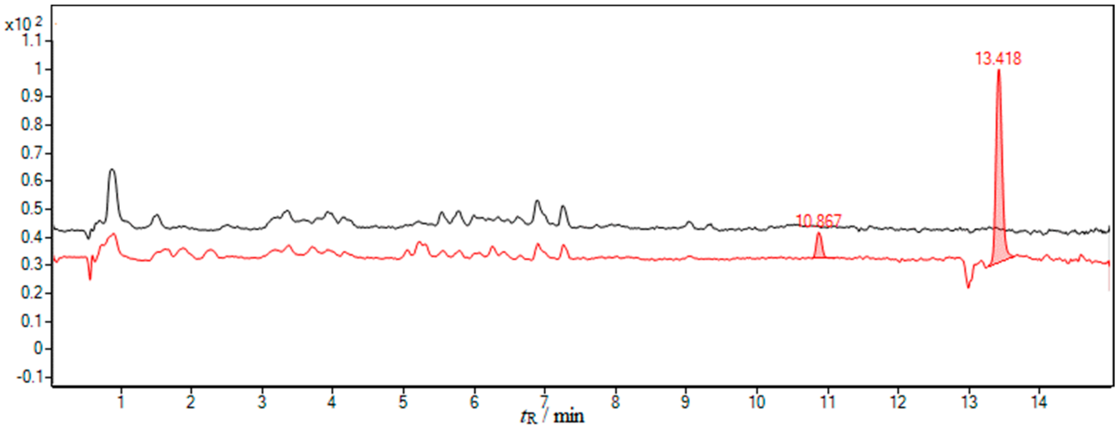


**Figure S12.** Total ion chromatogram of pure HP-β-CD (—) and NAB:HP-β-CD after the hydrolytic degradation in acidic media (—).

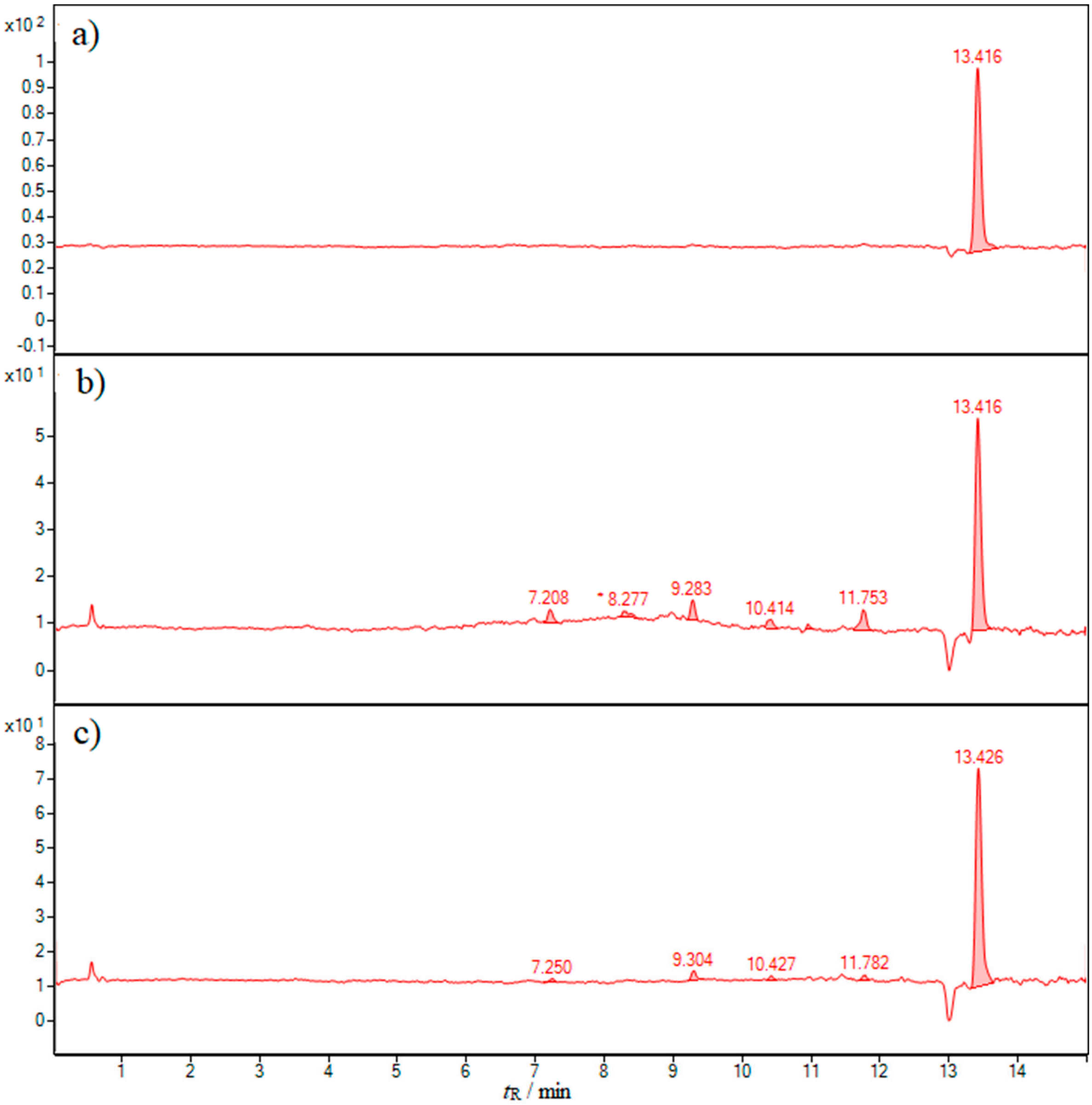


**Figure S13.** Total ion chromatograms of a) NAB, b) NAB-HP-β-CD, and c) NAB-SBE-β-CD under the oxidative degradation.

**Table S9.** MS/MS spectrum of degradation product DP 2 ($t_R$ = 7.2 min) at collision potential of 10 V.

| **Measured *m*/*z*** | **Rel. intensity / %** | **Assignation** |
|---|---|---|
| 237.0747 | **-** | $[M+H]^+$ |
| 219.0643 | **100** | $[C_{12}H_{10}O_4]^+$ |
| 161.0221 | 3.56 | $[C_9H_5O_3]^+$ |

**Table S10.** MS/MS spectrum of degradation product DP 3 ($t_R$ = 7.2 min) at collision potential of 10 V.

| **Measured *m*/*z*** | **Rel. intensity / %** | **Assignation** |
|---|---|---|
| 293.1023 | 6.22 | $[M+H]^+$ |
| 275.0919 | 33.26 | $[C_{15}H_{15}O_5]^+$ |
| 261.0754 | 7.33 | $[C_{14}H_{13}O_5]^+$ |
| 247.0964 | 11.30 | $[C_{14}H_{15}O_4]^+$ |
| 243.0653 | **100** | $[C_{14}H_{11}O_4]^+$ |
| 233.0807 | 15.22 | $[C_{13}H_{13}O_4]^+$ |
| 231.1016 | 14.07 | $[C_{14}H_{15}O_3]^+$ |
| 219.1015 | 40.98 | $[C_{13}H_{15}O_3]^+$ |
| 215.0700 | 8.14 | $[C_{13}H_{11}O_3]^+$ |

**Table S11.** MS/MS spectrum of degradation product DP 4 ($t_R$ = 8.8 min) at collision potential of 10 V.

| **Measured *m*/*z*** | **Rel. intensity / %** | **Assignation** |
|---|---|---|
| 251.0902 | **-** | $[M+H]^+$ |
| 233.0798 | 57.96 | $[C_{13}H_{13}O_4]^+$ |
| 219.0645 | **100** | $[C_{12}H_{11}O_4]^+$ |

**Table S12.** MS/MS spectrum of degradation product DP 5 ($t_R$ = 9.0 min) at collision potential of 10 V.

| **Measured *m*/*z*** | **Rel. intensity / %** | **Assignation** |
|---|---|---|
| 263.0902 | 7.78 | $[M+H]^+$ |
| 235.0957 | 1.59 | $[C_{14}H_{12}O_5]^+$ |
| 203.0686 | **100** | $[C_{12}H_{11}O_3]^+$ |

**Table S13.** MS/MS spectrum of degradation product DP 6 ($t_R$ = 9.3 min) at collision potential of 10 V.

| **Measured *m*/*z*** | **Rel. intensity / %** | **Assignation** |
|---|---|---|
| 277.1054 | 11.30 | $[M+H]^+$ |
| 245.0798 | 3.53 | $[C_{14}H_{13}O_4]^+$ |
| 227.0689 | 4.09 | $[C_{14}H_{11}O_3]^+$ |
| 203.0695 | **100** | $[C_{12}H_{11}O_3]^+$ |

**Table S14.** MS/MS spectrum of degradation product DP 7 ($t_R$ = 9.5 min) at collision potential of 10 V.

| **Measured *m*/*z*** | **Rel. intensity / %** | **Assignation** |
|---|---|---|
| 217.0854 | **100** | $[M+H]^+$ |
| 159.0432 | 5.54 | $[C_{10}H_7O_2]^+$ |

**Table S15.** MS/MS spectrum of degradation product DP 8 ($t_R$ = 10.4 min) at collision potential of 10 V.

| **Measured *m*/*z*** | **Rel. intensity / %** | **Assignation** |
|---|---|---|
| 261.1106 | **100** | $[M+H]^+$ |
| 229.0848 | 68.99 | $[C_{14}H_{13}O_3]^+$ |
| 203.0695 | 56.17 | $[C_{12}H_{11}O_3]^+$ |
| 201.0900 | 52.91 | $[C_{13}H_{13}O_2]^+$ |
| 171.0429 | 17.17 | $[C_{11}H_7O_2]^+$ |

**Table S16.** MS/MS spectrum of degradation product DP 9 ($t_R$ = 11.0 min) at collision potential of 10 V.

| **Measured *m*/*z*** | **Rel. intensity / %** | **Assignation** |
|---|---|---|
| 245.0773 | 10.40 | $[M+H]^+$ |
| 227.0688 | 51.69 | $[C_{14}H_{11}O_3]^+$ |
| 209.0576 | 14.94 | - |
| 201.0897 | 4.83 | $[C_{13}H_{13}O_2]^+$ |
| 199.0744 | 19.76 | $[C_{13}H_{11}O_2]^+$ |
| 185.0601 | 16.02 | $[C_{12}H_9O_2]^+$ |
| 183.0792 | **100** | $[C_{13}H_{11}O]^+$ |

**Table S17.** MS/MS spectrum of degradation product DP 10 ($t_R$ = 11.8 min) at collision potential of 10 V.

| **Measured *m*/*z*** | **Rel. intensity / %** | **Assignation** |
|---|---|---|
| 259.0949 | 15.79 | $[M+H]^+$ |
| 241.0846 | 55.49 | $[C_{14}H_{11}O_3]^+$ |
| 227.0691 | 15.87 | $[C_{14}H_{11}O_2]^+$ |
| 209.0585 | 71.28 | $[C_{13}H_{11}O_2]^+$ |
| 199.0741 | 10.48 | $[C_{14}H_{13}O]^+$ |
| 197.0953 | **100** | $[C_{13}H_{11}O]^+$ |

**Table S18.** MS/MS spectrum of degradation product DP 11 ($t_R$ = 11.8 min) at collision potential of 10 V.

| **Measured *m*/*z*** | **Rel. intensity / %** | **Assignation** |
|---|---|---|
| 291.0857 | **100** | $[M+H]^+$ |
| 259.0595 | 54.86 | $[C_{14}H_{11}O_5]^+$ |
| 231.0643 | 4.72 | $[C_{13}H_{11}O_4]^+$ |
| 203.0697 | 10.16 | $[C_{12}H_{11}O_3]^+$ |

## 3.2. Photostability study

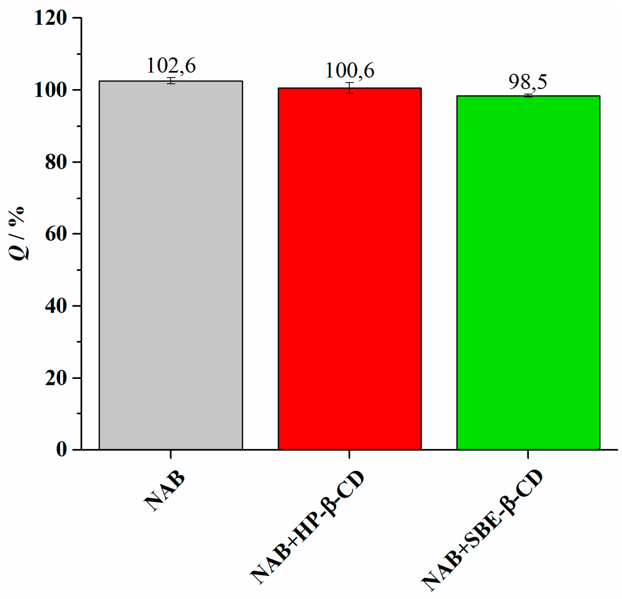


**Figure S14.** NAB content in sample of pure NAB and in NAB-HP-β-CD and NAB-SBE-β-CD samples prepared by grinding subjected to photostability studies

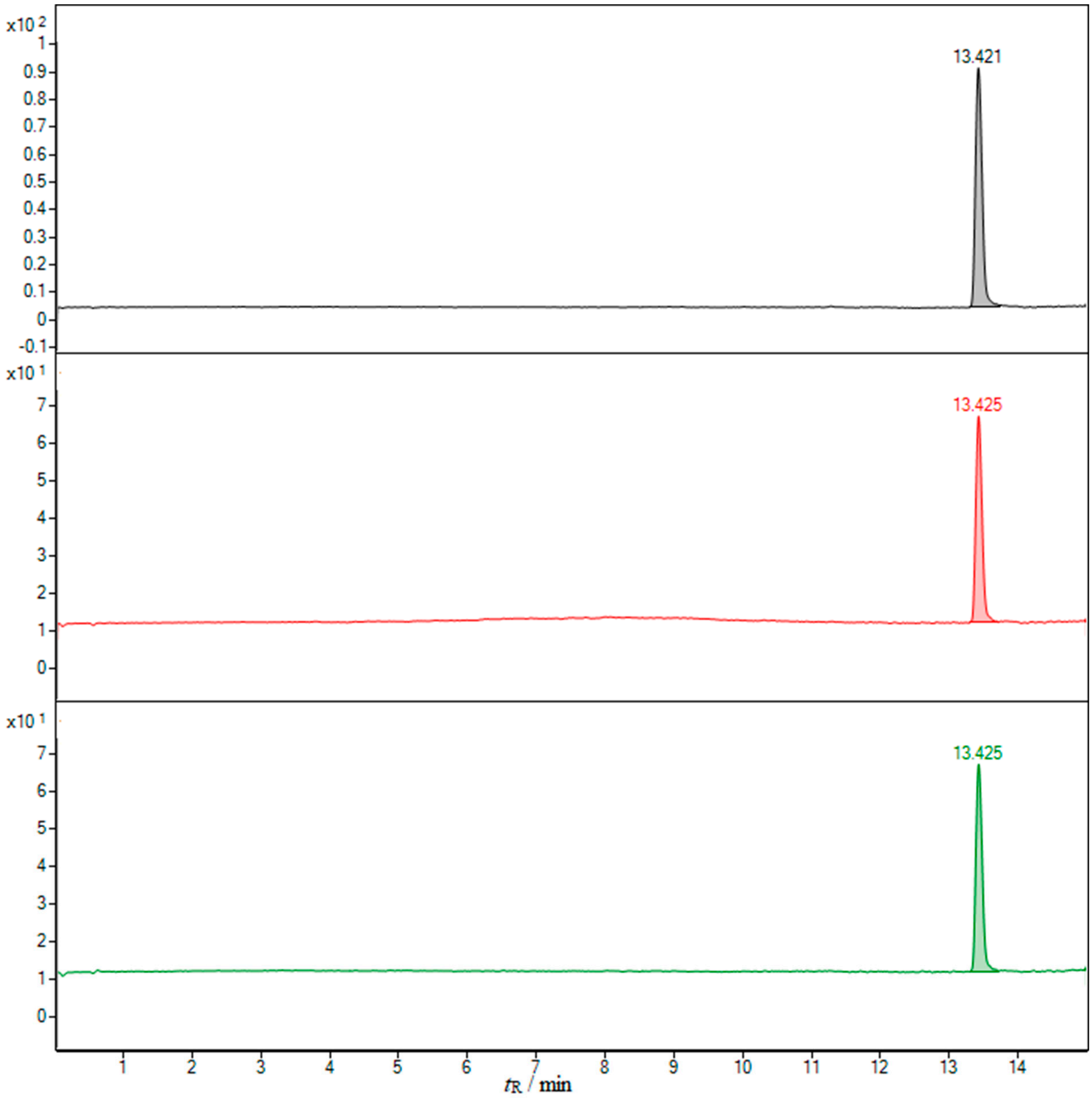


**Figure S15.** Total ion chromatograms of NAB (—), NAB-HP-β-CD (—), and NAB-SBE-β-CD (—) samples after subjecting to photostability studies.

### 3.3. Long-term stability study

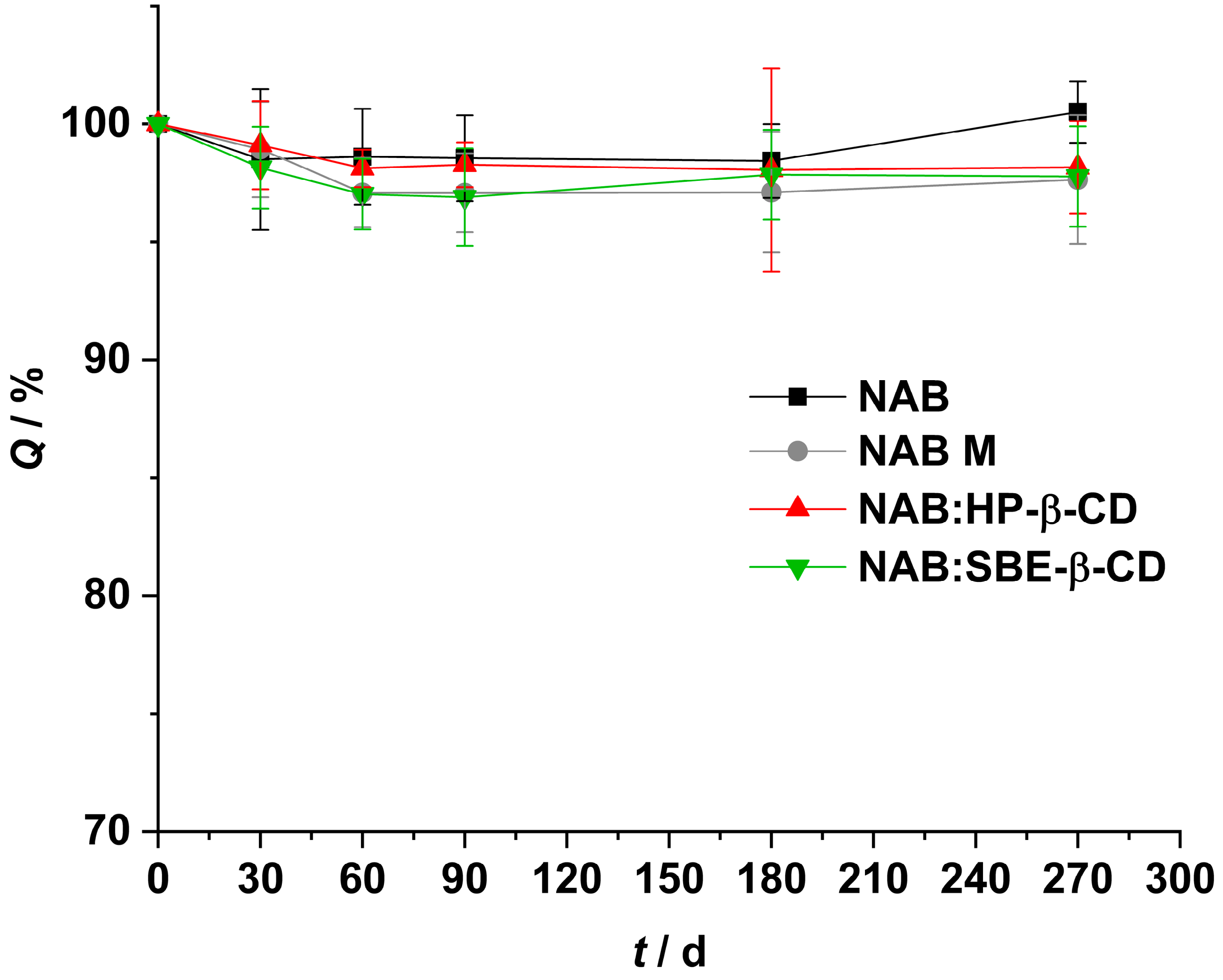


**Figure S16.** NAB content in samples of pure NAB, grinded NAB and in NAB-HP-β-CD and NAB-SBE-β-CD samples prepared by grinding during the long-term stability studies for 9 months.

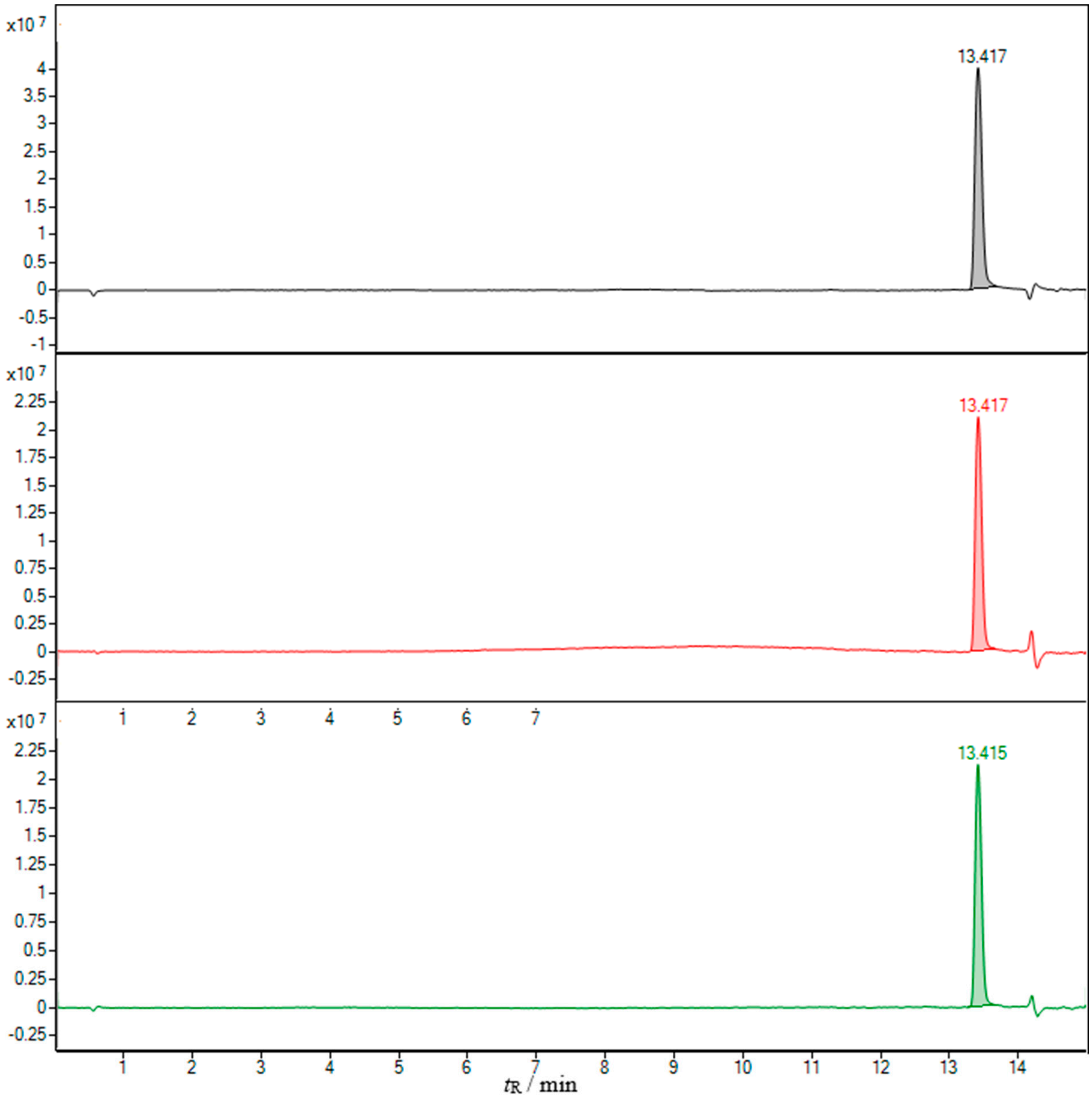


**Figure S17.** Total ion chromatograms of NAB (—), NAB-HP-β-CD (—), and NAB-SBE-β-CD (—) samples after the period of 9 months of long-term stability studies.
